# Conserved Cdk inhibitors show unique structural responses to tyrosine phosphorylation

**DOI:** 10.1016/j.bpj.2022.05.024

**Published:** 2022-05-25

**Authors:** Jacob B. Swadling, Tobias Warnecke, Kyle L. Morris, Alexis R. Barr

**Affiliations:** 1 Institute of Clinical Sciences, Imperial College London, London, United Kingdom; 2 MRC London Institute of Medical Sciences, London, United Kingdom

## Abstract

Balanced proliferation-quiescence decisions are vital during normal development and in tissue homeostasis, and their dysregulation underlies tumorigenesis. Entry into proliferative cycles is driven by Cyclin/Cyclin-dependent kinases (Cdks). Conserved Cdk inhibitors (CKIs) p21^Cip1/Waf1^, p27^Kip1^, and p57^Kip2^ bind to Cyclin/Cdks and inhibit Cdk activity. p27 tyrosine phosphorylation, in response to mitogenic signaling, promotes activation of CyclinD/Cdk4 and CyclinA/Cdk2. Tyrosine phosphorylation is conserved in p21 and p57, although the number of sites differs. We use molecular-dynamics simulations to compare the structural changes in Cyclin/Cdk/CKI trimers induced by single and multiple tyrosine phosphorylation in CKIs and their impact on CyclinD/Cdk4 and CyclinA/Cdk2 activity. Despite shared structural features, CKI binding induces distinct structural responses in Cyclin/Cdks and the predicted effects of CKI tyrosine phosphorylation on Cdk activity are not conserved across CKIs. Our analyses suggest how CKIs may have evolved to be sensitive to different inputs to give context-dependent control of Cdk activity.

## Significance

Cell proliferation is driven by the activation of Cyclin/Cyclin-dependent kinases (Cdks). Cyclin/Cdk activity is tightly regulated to prevent unscheduled activation, which can promote tumorigenesis. Three conserved inhibitors bind to Cyclin/Cdks: p21, p27, and p57. Inhibitor activity is modified by their phosphorylation downstream of pro-proliferative signaling. We systematically investigate the effect of inhibitor phosphorylation on the structure of Cyclin/Cdk/inhibitor complexes and the implications for Cdk activity. Despite structural similarity, the three inhibitors respond in non-intuitive ways to phosphorylation, leading to diverse effects on Cdk activity, providing insight into how proliferation can be regulated in a context-dependent manner. Scrutinizing Cyclin/Cdk/inhibitor interactions also contributes to our understanding of the mechanism of action of clinically relevant Cdk4/6 inhibitors, which may act by displacing Cdk inhibitors.

## Introduction

During development and in tissue homeostasis, the controlled release of cells from a quiescent (resting, G0) state into proliferative cycles is vital to achieve and maintain a healthy state. Dysregulation of these pathways underpins abnormal development and tumorigenesis. Cell proliferation can be induced by multiple signals, including mitogenic growth factors, the extracellular matrix, and cell-cell contacts. Despite differences in upstream signaling, common to signals that promote proliferation is the downstream activation of Cyclin-dependent kinases (Cdks) that drive entry into and through the cell cycle (G1, S, G2, and mitosis) (1). How context-dependent signals are translated into changes in Cdk activity is not fully understood.

**Figure 1:**
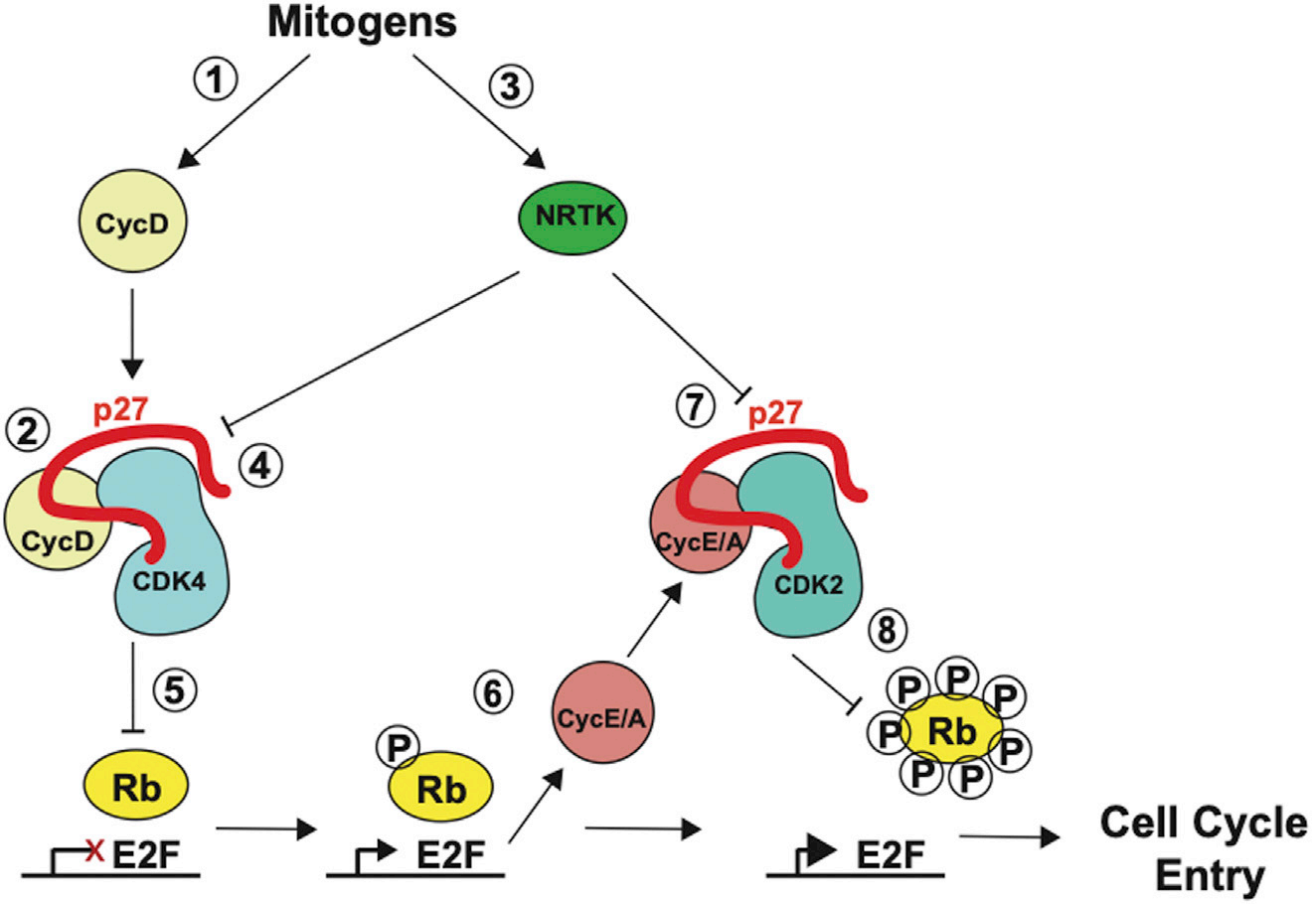
Role of tyrosine phosphorylation in initiating cell-cycle entry. Mitogen stimulation of quiescent cells leads to CyclinD (CycD) expression (*1*) which binds to Cdk4, aided by p27 (*2*). Mitogens also activate non-receptor tyrosine kinases (NRTK) (*3*) that phosphorylate p27^Y74^ in CyclinD/Cdk4 complexes (*4*). This leads to partial activation of CyclinD/Cdk4 that can phosphorylate the transcriptional repressor protein, Rb (*5*). Rb phosphorylation allows activation of the E2F transcription factor, which drives CyclinE and A expression (*6*). CyclinE and A bind to Cdk2, but Cdk2 activity can be inhibited by p27 binding. Non-receptor tyrosine kinases phosphorylation of p27^Y88^ (*7*) releases the $3_{10}$ helix from the Cdk2 active site, activating Cdk2. CyclinE,A/Cdk2 further phosphorylate Rb (*8*), leading to full activation of the E2F transcriptional program, which promotes entry into S phase. To see this figure in color, go online.

**Figure 2:**
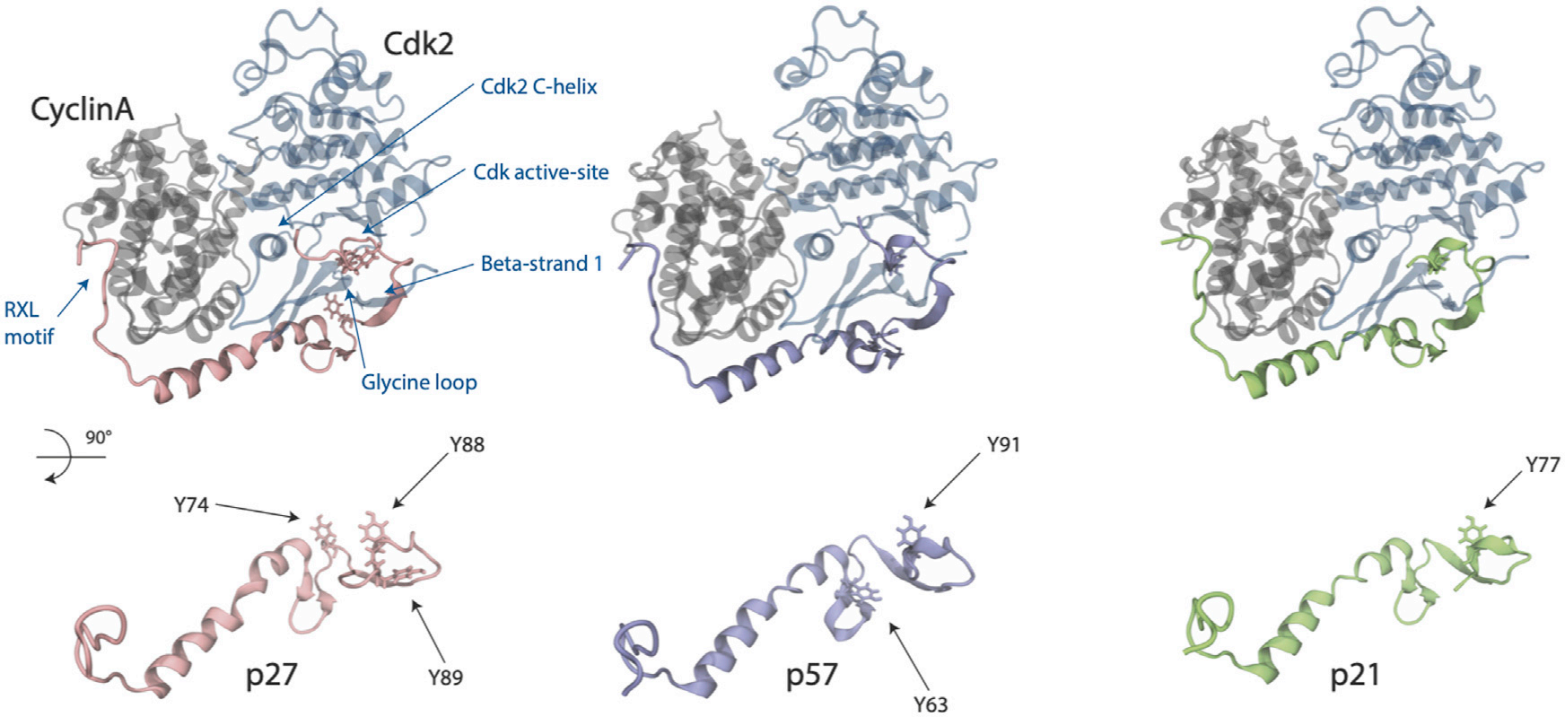
Starting structures of Cdk2 (*blue*), Cyclin A (*gray*) in complex with p27 (*pink*), p57 (*purple*), and p21 (*green*) shown as a secondary-structure representation taken after minimization and equilibration. The RXL motif in p27, and the active site, first beta strand, C-helix, and glycine loop in Cdk2 are all labeled. In the bottom half of the figure, the three proteins are rotated, and tyrosine residues are depicted in a liquorice representation with residue numbers highlighted. To see this figure in color, go online.

Cdks are subject to layers of regulation to ensure quiescent cells only enter the cell cycle in response to appropriate environmental cues (2). Cdk activation requires Cyclin binding and T-loop phosphorylation in the Cdk-activating segment by Cdk-activating kinase (CAK). Mitogenic signaling promotes Cyclin D expression, which binds Cdk4 (or Cdk6) to initiate progression from G0 into G1 (Fig. 1). Downstream of CyclinD/Cdk4, Cyclin E then Cyclin A expression activates Cdk2 and promotes the transition of cells from G1 into S phase (DNA replication). Cdks can be inhibited via Wee1— and Myt1—mediated phosphorylation, which is opposed by Cdc25 phosphatases (3,4). Two families of Cdk inhibitors (CKIs) also inhibit Cdk activity: the INK4 and Cip/Kip proteins. Cip/Kip family members (p21^Cip1/Waf1^, p27^Kip1^, and p57^Kip2^) are intrinsically disordered proteins that fold upon binding to Cyclin/Cdk dimers to inhibit Cdk activity (5, 6, 7, 8, 9, 10). Cip/Kips can inhibit Cdks in multiple ways (11, 12, 13). In both Cdk2 and Cdk4 complexes, Cip/Kip proteins physically block the substrate-binding site (via their RXL motif) on the Cyclin subunit and destabilize ATP binding by remodeling the N lobe of the Cdk—displacing the first beta strand and removing the glycine loop that normally binds the ATP phosphates (Fig. 2). In Cdk2, insertion of the Cip/Kip $3_{10}$ helix into the ATP-binding pocket also mimics and blocks ATP binding. Coordination of Cdk-activating and -inhibiting activities is required to prevent unscheduled Cdk activity and promote timely cell-cycle entry.

Mitogenic signaling not only stimulates CyclinD expression but also initiates the removal of p27 (Fig. 1). Levels of p27 protein are high in quiescent cells and help maintain the quiescent state (14, 15, 16). p27 binding to CyclinD/Cdk4 releases the activation segment of Cdk4 to permit CAK phosphorylation of CyclinD/Cdk4 (13). However, the CAK-phosphorylated CyclinD/Cdk4/p27 remains inactive. Mitogen signaling activates non-receptor tyrosine kinases (NRTKs), including the proto-oncogenes Src, Abl, Yes, Lyn, and Brk/PTK6. These tyrosine kinases phosphorylate p27 on one or more of three tyrosine residues that reside in the Cdk-binding domain: Y74 (Src, Yes, and Brk), Y88 (all NRTKs), or Y89 (Abl, Lyn, and Brk) (17,18). For Cdk4, p27^Y89^ phosphorylation promotes activation of Cdk4 by CAK (19), and Y74 phosphorylation destabilizes the interaction between p27 and CyclinD/Cdk4, allowing Cdk4 to become active and initiate cell-cycle entry. For Cdk2, phosphorylation of Y88 in p27 leads to the ejection of the $3_{10}$ helix from the Cdk2 active site, partially activating Cdk2 (20). This initiates a positive feedback loop where CyclinA/Cdk2 intramolecularly phosphorylates p27^T187^, which promotes p27 recognition and ubiquitination and subsequent degradation by SCF^Skp2^ (21).

All three Cip/Kips display overlapping patterns of tissue expression (22), and, although divergent in their C-termini, are highly conserved within their N-terminal domains (23,24)—the region that mediates Cyclin/Cdk binding and that is sensitive to tyrosine phosphorylation. This suggests that all three interact with Cyclin/Cdks in a similar way, although this remains to be tested. Indeed, NRTKs can phosphorylate all three proteins in vitro (25). The number and distribution of tyrosine sites in the N-termini of the Cip/Kip proteins differ, with p27 having three sites (Y74, Y88, and Y89), p57 two sites (Y63 and Y91), and p21 just one (Y77) suggesting that different Cip/Kips might show unique responses to tyrosine phosphorylation (Fig. 2). Recent crystal structures comparing p21 and p27 bound to CyclinD/Cdk4 indicate that the lack of a p27^Y74^ equivalent in p21 could be a key difference between how p21 and p27 regulate Cdk4 activity, such that even after NRTK activation, p21 would remain a CKI (13). This may explain how p21 is able to induce quiescence after intrinsic DNA damage even in the presence of continued growth factor signaling (26, 27, 28). Systematic analyses of the interactions between Cyclin/Cdk dimers and the three Cip/Kips, and the role of tyrosine phosphorylation in regulating Cdk4 and Cdk2 activity are lacking, hampering our understanding of how Cdk activity is regulated in different contexts. In addition, in p27 and p57, where multiple tyrosine phosphorylation sites exist, it is not known if a hierarchy of phosphorylation exists or if cooperativity exists between sites.

Classical molecular dynamics (cMD) and accelerated molecular dynamics (aMD) simulations have been applied to investigate the folding and binding of p27 in solution (6) and revealed how p27 exhibits intrinsic flexibility such that buried tyrosine residues can be phosphorylated, promoting the ejection of the $3_{10}$ helix from the Cdk2 active site, initiating CyclinA/Cdk2 activation (29, 30, 31). Whether similar flexibility exists in p57 and p21 is unknown. In the absence of available crystal structures of all the possible Cyclin/Cdk/Cip/Kip combinations, with different combinations of tyrosine phosphorylation states, we have used fully atomistic simulations to systematically compare the binding of p21, p27, and p57 with CyclinA/Cdk2 and CyclinD/Cdk4 complexes and to probe the structural changes mediated by single and multi-site tyrosine phosphorylation across all three Cip/Kips in the regulation of Cdk activity. Despite seemingly conserved mechanisms of Cip/Kip binding to Cyclin/Cdk dimers, we show that p21 binding to CyclinA/Cdk2 has a more significant effect on the disruption of hydrogen bonding at the Cyclin/Cdk interface, suggesting an additional mechanism by which p21 could influence Cdk2. Our simulations reveal that tyrosine phosphorylation of the Cip/Kips induces non-conserved, non-intuitive structural changes, suggesting that it is insufficient to extrapolate from what we know regarding p27 phosphorylation to p21 and p57 function. We also show how Cip/Kip binding to CyclinA/Cdk2 can block phosphorylation by Wee1 and Myt1 kinases, imposing an order of events on Cdk2 regulation. Together, our analyses show how p21, p27, and p57 have evolved distinct structural responses to tyrosine phosphorylation that would enable cells to respond specifically to context-dependent signaling.

## Materials and methods

### Model construction

The starting structure of p27/Cdk2/CyclinA was taken from the crystal structure (1JSU) (11), of resolution 2.30 Å. The homology models of p21- and p57-bound ternary complexes were constructed using UniProt sequences with the SWISS-MODEL tool and aligned to the 1JSU crystal structure (32). Models were constructed of doubly protonated phosphorylated tyrosine in each model as this is the predicted protonation state at pH 7.4 derived using PROPKA (33). Cdk T14 was phosphorylated in models 3 and 4 (see Table S1 for details). For p27, Y88/Y89/Y74 were phosphorylated and each combination therein. For p57, Y91/Y63 were phosphorylated and for p21, Y77 was phosphorylated. All models were parameterized using Amber14SB, the most up-to-date potentials for canonical proteins (34). Missing potentials for the phosphorylated tyrosine/threonine residues were provided by Homeyer et al. (35) in the same manner used in recent studies describing the interaction of phosphorylated p27 (29). Models were solvated with 14 Å of TIP3P water and neutralized with NaCl. Cdk4/CyclinD/CKI models were similarly built from the crystal structure (6P8E) (13), of resolution 2.30 Å. As the $3_{10}$ terminal region of p27 was not observed as an ordered structure in the electron density map with Cdk4/CyclinD, this was added to make a direct comparison with the Cdk2/CyclinA models.

### Molecular dynamics

Energy minimization was performed for 2000 steps using combined steepest-descent and conjugate-gradient methods. Following minimization, 20 ps of cMD was performed in the constant temperature, constant volume ensemble using a Langevin thermostat (36) to regulate the temperature as we heated it up from 0 to 300 K. Following the heat-up phase, we performed 500 ns of cMD in the isobaric/isothermal (constant temperature, constant pressure) ensemble using the Berendsen barostat (37) to maintain constant pressure and an integration step of 2 fs. At the beginning of the constant temperature, constant pressure production phase, velocities were randomly selected from a Maxwell Boltzmann distribution. All simulations were performed using GPU (CUDA) v.18.0.0 of PMEMD (38, 39, 40) with long-range electrostatic forces treated with particle-mesh Ewald summation (41), with a cut-off of 9 Å. Using information gathered from the cMD simulations regarding the time-averaged potential energy of the system and dihedral energy, we performed aMD (42) on all models. The aMD method was employed to identify transitions to conformational states that might otherwise be inaccessible within the timescale used in cMD. A full description of the aMD method can be found in the supporting material. We have shown that these simulations are stable over 500 ns of non-biased simulation. We ran three replicas of the classical MD for a number of models, as described in Table S1. The potential energy barrier is sufficiently high, as the trajectory does not follow the transition path out of the minima at 300 K over 1500 ns. The additional sampling was not significant, and we continued with 500 ns in subsequent simulations.

We simulated a total of 20 molecular systems using cMD and aMD, totalling over 20 μs of simulation, of which the details are given in Table S1. Y2P denotes a twice-protonated phosphorylation of tyrosine, and T2P denotes a twice-protonated phosphorylation of threonine, e.g., Y2P88 indicates the tyrosine residue at residue number 88 has been phosphorylated with two protonation sites (total charge of -2), as per the nomenclature in (35).

The per-residue decomposition of the binding free energy was calculated using the molecular mechanics Poisson-Boltzmann surface area method (43), where the free energy of solvation was approximated using the Poisson-Boltzmann implicit water model. The secondary structure was identified using the DSSP method (44) with cpptraj analysis tools (45). The hydrogen-bonding criteria we used was an angle of 135° and a distance of 3.0 Å, and we consider Oxygen, Fluorine and Nitrogen heavy atoms. The measure of dissociation was based on the center of mass of Y88. The root-mean-squared deviation (RMSD) was calculated on C-alpha atoms. We calculated the best-fit RMSD that each structure is rotated and translated so as to minimize the RMSD to the reference structure (the starting structure).

### Identification of Cip/Kip family members and phylogenetic reconstruction

We searched OrthoDB v.10 (https://www.orthodb.org) for p21, p27, and p57 based on their corresponding UniProt identifiers (UniProt: P38936, P46527, and P49918, respectively) and downloaded the ortholog group that contained all three proteins (1595421) at the Eukaryota level (1595421at2759). Orthologs were then aligned using MAFFT (mafft-linsi). The resulting protein-level alignment was used to compute a maximum likelihood phylogeny using RAxML (v.8.1.16 -f a -m PROTCATAUTO).

## Results

### Tyrosine phosphorylation of Cip/Kips is conserved across eukaryotes

Tyrosine phosphorylation plays key roles in cellular signaling, including in cell cycle control. Proteins regulating the cell cycle are highly conserved across eukaryotes. Therefore, to investigate the conservation of tyrosine phosphorylation and its effects on the structure of Cip/Kip inhibitors and bound Cyclin/CDK complexes, we first took an evolutionary approach to determine the gain and loss of these key signaling hubs. p21, p27, and p57 bear distinct signatures of confirmed and putative phosphorylation sites. Focusing on tyrosine residues known to be important for p27 function (Y74, Y88, and Y89), we note that only Y88 is almost universally conserved across vertebrate Cip/Kip family members (Fig. S1 *a*). The notable exceptions are p21 orthologs in non-mammalian tetrapods, which all lack Y88 (Fig. S1 *a* and *b*). However, Y88 remains present in p20 (CDKN1D), perhaps suggesting that mammalian p21 functionality might be partitioned between p21 and p20 in these species (Fig. S1 *a*). Y74 is generally present in p27 orthologs, and the single Cip/Kip copy in non-vertebrates, and is particularly well conserved in mammals but is largely absent from p21 and p57. Finally, Y89 is restricted to eutherian mammals, i.e., absent from marsupials and monotremes as well as non-mammalian vertebrates. Altogether, our analyses suggest that Y88 phosphorylation may have similar roles in regulating the functions of all Cip/Kips across eukaryotes, whereas other tyrosine phosphorylation sites may play more Cip/Kip-specific roles.

Each paralogous group also contains several, often well-conserved, residues that could, in principle, be subject to dynamic phosphorylation (Fig. S1 *b*). We restricted our analysis to S/T-P sites, which can be phosphorylated by Cdks, MAP kinases, and GSK3B, all kinases implicated in mitogenic signaling (Fig. 1). Some focal residues (e.g., the equivalent of Y74 in p27 in p21 and p57) may be compensated by the presence of other residues that are not in an orthologous position but close by, such as Y63 in p57 and T57 in p21, each of which is highly conserved (Fig. S2). We will come back to the potential significance of these residues in our later analyses.

### Hydrogen-bonding networks reveal differences in intermolecular contacts between CyclinA, Cdk2, and CKIs

**Figure 3:**
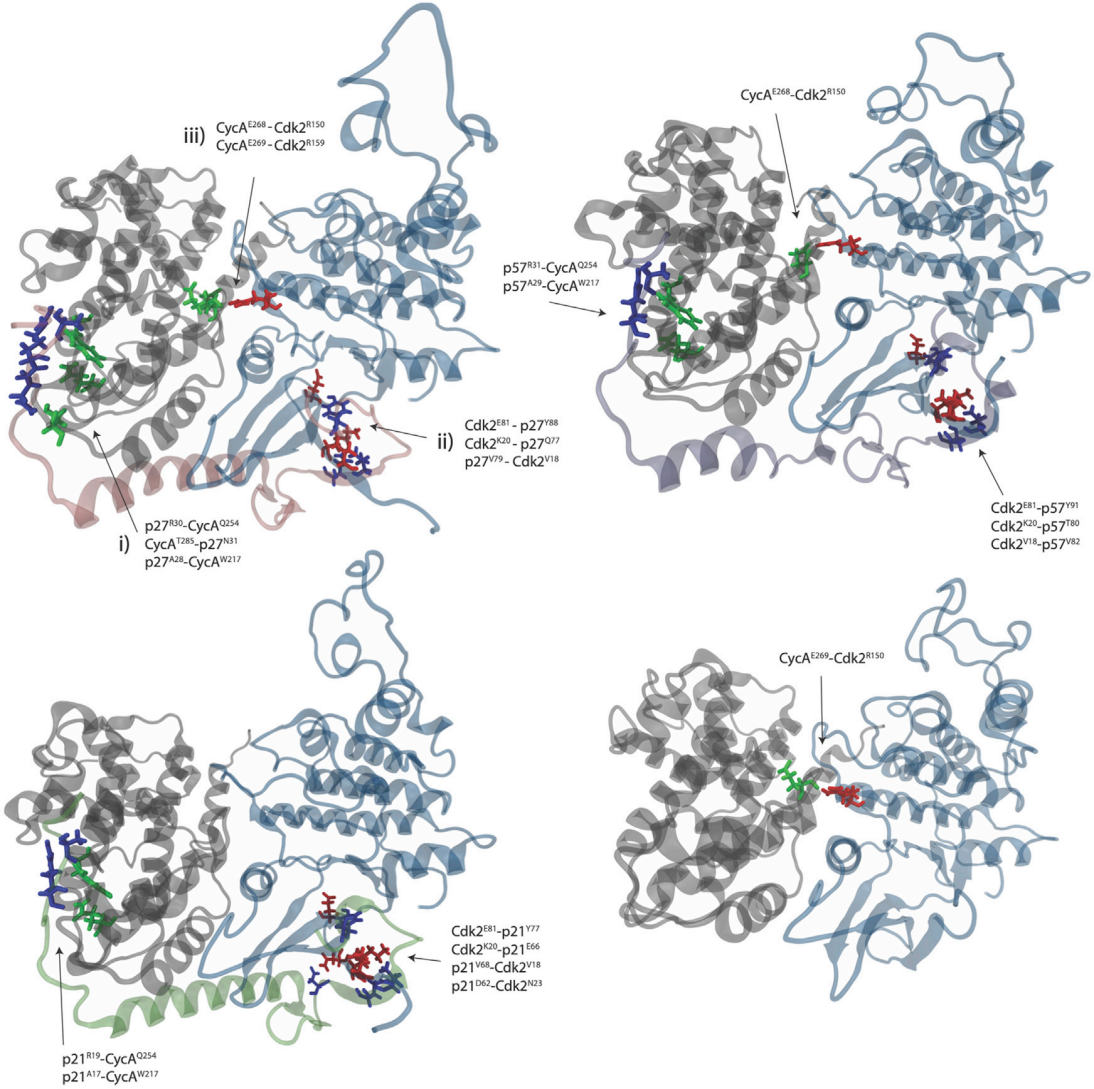
Structures taken from the final frame of each cMD simulation at 500 ns. Intermolecular hydrogen bonds were calculated over the last 400 ns of simulation, and contacts between molecules with a fraction lifetime above 0.5 are displayed on the full structure in the order of hydrogen-bond acceptor first and donor second. This simplifies the major, long-lived intermolecular contacts between Cdk2, CyclinA, and inhibitor. Residues are colored according to the molecular fragment they belong to: gray is CyclinA, blue is Cdk2, and the CKI is shown in pink (p27), green (p21), or purple (p57). The RXL motif is p27^R30,N31,L32^; p21^R19,R20,L21^; p57^R31,S32,L33^. To see this figure in color, go online.

Before investigating the role of tyrosine phosphorylation on Cyclin/CDK/Cip/Kip interactions and CDK activity, we sought to understand the effects on the CyclinA/Cdk2 dimer on binding of the different Cip/Kip proteins. The structure of CyclinA/Cdk2 bound to p27 has been solved (11) but has not been for p21 or p57. To understand the key contacts made between Cyclin, Cdk, and the inhibitor in the ternary complex, we derived homology models for CyclinA/Cdk2/p21 and CyclinA/Cdk2/p57 (Fig. 2). We performed cMD simulations and calculated all intermolecular hydrogen bonds over the final 400 ns of trajectories. Contacts that had a life-time fraction above 0.5 were recorded (Tables S2–S5) and are superimposed upon the final frame of each trajectory (Fig. 3). The selected residues represent the major contacts between the three molecules in the ternary complex, which we compare with the CyclinA/Cdk2 dimer.

The hydrogen bonding network reveals three significant areas of interaction between the three molecules: 1) between Cdk2 and CyclinA, mediated by hydrogen bonding between Cdk2 R150 and CyclinA E268/E269, 2) between Cdk2 and CKI, predominantly through interactions between Cdk2 E81, K20, and V18 and the CKI, and 3) between CyclinA and CKI, via CyclinA Q254 and W217 and the CKI RXL motif (Fig. 3). While these are homology models and not crystal structures, we are confident that they are representative of the Cip/Kip-bound CyclinA/Cdk2 dimer, as these key contacts are maintained across all three inhibitor-bound complexes. Moreover, the hydrogen-bonding network revealed by our simulations echoes that observed in the Cdk2/CyclinA/p27 crystal structure (11). The simulation of Cdk2/CyclinA/p27 correctly identifies the interaction of the rigid end coil of p27 with the shallow CyclinA groove through the RXL motif and adjacent residues in p27 (A28) with CyclinA Q254, T285, and W217, as shown in Fig. 3 (also see Fig. 3 in reference (11) for crystal contacts). Our trajectories also corroborate the importance of binding between p27 Y88 and Cdk2 E81 in the ATP-binding site, as reported by Russo et al. (11) (see Tables S2–S5). Residue R150 in Cdk2 is in the N-terminal region of the T loop and forms hydrogen bonds to T160, according to (11). Furthermore, we observe the hydrogen bond between Cdk2 R150 and CyclinA E268 to be a dominant interaction in the dimer when p27 is bound. We observe changes in H-bonding at the CyclinA/Cdk2 interface that p27 binding induces, in particular at intermolecular contacts between CyclinA and Cdk2, through two glutamic acid-arginine residue pairs, E268-R150 and E269-R159, consistent with structural changes induced in Cdk2 by p27 binding (11).

Despite the three significant areas of interaction stated previously persisting in all CKI-bound complexes, we observe notable differences in the intermolecular interactions between molecules. We measure the fraction of the lifetime of a hydrogen bond as a probability, e.g., P(bound/unbound). If the contact is present for 1 ns or longer, it is deemed to be bound, and if not, unbound. When p21 is bound, we observe a reduction in the fraction of the lifetime of the hydrogen bond between CyclinA and Cdk2 (0.69–0.39), namely CycA^E268^ and Cdk2^R150^, compared with bound p27 (0.56) and p57 (0.57). Furthermore, when p21 binds CyclinA/Cdk2, we see the formation of a long-lived hydrogen bond between Cdk2^N23^ and p21^D62^, which we do not observe in the corresponding positions in p27 or p57. This increased affinity at close proximity to the ATP-binding site moves Cdk2 in such a way that we observe the loss of long-lived interactions between the CyclinA/Cdk2 interface. This could represent an additional mechanism by which p21 might influence Cdk2 activity that differs to p27 and p57.

Indeed, while not conserved at the sequence level, Y63 in p57 binds to the CyclinA/Cdk2 complex in a position near to Y74 in p27 and may be expected to play a similar role by disrupting the interface with Cdk2 when phosphorylated by NRTKs rather than because the residues are equivalent (Fig. 2). For this reason, although p57^Y63^ binds in an equivalent position to p27^F62^ in the trimer, we consider p57^Y63^ as analogous to p27^Y74^. Moreover, while not a tyrosine residue and so not explicitly considered in our analyses, ERK, JNK, and GSK3 phosphorylation of p21^T57^ decreases p21 stability by promoting its degradation (shown in Fig. S2) (46, 47, 48, 49, 50).

Our homology modeling and calculation of intermolecular hydrogen bonding in the CyclinA/Cdk2/CKI trimers show conserved inhibition mechanisms across all Cip/Kips through RXL motif docking and occlusion of the Cdk2 active site. We observe differences in the key contacts made between the inhibitors and the dimer, i.e., p27 RXL motif with Cyclin A and p21 with the Cdk2 active site. These differences may be significant in conferring specificity in CKI inhibition and how individual CKIs respond to upstream signaling.

### Tyrosine phosphorylation of conserved Cip/Kips induces distinct conformational changes in the CyclinA/Cdk2/CKI complex

Having established the conservation of key tyrosine residues in Cip/Kip inhibitors and determined differences in how unphosphorylated CKIs interact with the CyclinA/Cdk2 dimer, we wanted to investigate the effect that phosphorylation of these sites would have on the structure of CyclinA/Cdk2/CKI trimers and, ultimately, Cdk2 activity. Tyrosine phosphorylation of p27 leads to conformational changes in both the bound Cyclin/Cdk complex and in p27 itself (30). A systematic investigation into how phosphorylation at each of the three tyrosine residues in p27 alone and in combination could affect these conformational changes, and thus if a potential p27 signaling hierarchy or “code” could exist, has yet to be performed. Similarly, whether conformational changes occur in p21 and p57 complexes when conserved tyrosines are phosphorylated remains hitherto unknown, making it difficult to predict the outcome of upstream signaling events on downstream cell-cycle outcomes.

**Figure 4:**
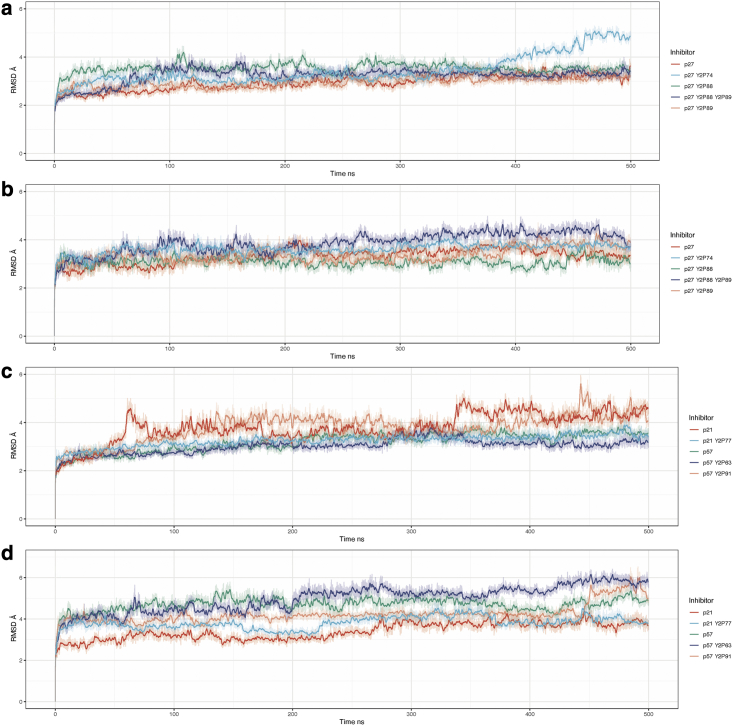
(*a* and *b*) Root-mean-squared deviation relative to the starting structure of (*a*) all CyclinA/Cdk2/p27 models and (*b*) the isolated p27 inhibitor from each model. (*c* and *d*) CyclinA/Cdk2 with (*c*) the p21/p57 partners and (*d*) the isolated p21/p57 molecules from each model. All models appear to equilibrate after a conservative 25 ns. All time-averaged data were taken with the initial 25 ns of data dropped. To see this figure in color, go online.

To investigate the dynamics of the different Cip/Kip-bound CyclinA/Cdk2 complexes and to identify which structural features warrant further investigation, we determined the RMSD of each model and that of the inhibitor alone (within the context of CyclinA/Cdk2 binding) in each state of tyrosine phosphorylation we have modeled, relative to the starting structure (Fig. 4). We show a single trajectory for each model over a 500 ns time course. It appears that from this general collective variable that each of the models has reached a steady state after 25 ns. To understand the time-averaged properties of the systems, we drop the first 25 ns from each of the three replicate simulations.

When p27^Y74^ is phosphorylated, p27 shows a significant deviation from the equilibrium average just before 400 ns (Fig. 4 *a*, *light blue*). As this is not seen in the RMSD of the inhibitor alone (Fig. 4 *b*), it appears that this deviation occurs in the CyclinA/Cdk2 dimer as a consequence of p27^Y74^ being phosphorylated. An alignment of trajectory frames taken at 500 ns for p27 and p27^Y74^, shown in Fig. S3, shows a conformational change in CyclinA of the N-terminal alpha helix at the CyclinA/Cdk2 interface that is shifted away from Cdk2 when Y74 is phosphorylated. By analyzing intermolecular hydrogen bonds over 250–500 ns for the p27^Y2P74^ complex, we see the establishment of longer-lasting hydrogen bonds upon Y74 phosphorylation between CyclinA E268 and F267 and Cdk2 R157 and R150. We see little deviation in Y88, Y89, or doubly phosphorylated Y88/Y89 p27 from the steady-state equilibrium in these classical MD simulations, indicating that these structures exhibit few conformational changes on these timescales (Fig. 4 *a*). Phosphorylation of Y88 is known to drive the ejection of the $3_{10}$ helix from the Cdk2 active site (29,31). However, this is a slow transition and unlikely to be visible in cMD simulations (31).

As the p21 and p57 sequences were templated on to the crystal structure for p27, it is not unexpected that they deviate more than that of p27 (compare Fig. 4 *b* and *d*). Unlike what we observed for p27, for p21, we see CyclinA/Cdk2/p21 jumping from one state to another, happening at around 60 and 340 ns (Fig. 4 *c*).These shifts seem to occur in the CyclinA/Cdk2 dimer, as they are not observed in the p21 inhibitor (Fig. 4 *d*). The jump between two RMSD states appears to be due to the two conformations of the Cdk2 N-lobe, as shown in Fig. 4 *a*. Phosphorylation of p21 Y77, which sits in the Cdk2 active site, abolishes these shifts between states (Fig. 4 *c*). Closer inspection of CyclinA/Cdk2 bound to p21 phosphorylated at Y77 shows long-lasting intermolecular hydrogen bonding between all three molecules (see Fig. 4 *b*). For p57 Y91 phosphorylation, which also sits in the Cdk2 active site, we see the opposite effect to that of p21 Y77 phosphorylation, where tyrosine phosphorylation promotes transitions between states, compared with the unphosphorylated protein (Fig. 4 *c*). This deviation appears to occur in p57 (Fig. 4 *d*) and in part represents the ejection of the $3_{10}$ helix from the Cdk2 active site when p57^Y91^ is phosphorylated.

Unlike p27^Y74^ phosphorylation, which appears to promote a change in the CyclinA/Cdk2 structure, phosphorylation of p57 at Y63 leads to a deviation in the structure of p57, not the CyclinA/Cdk2 complex (Fig. 4 *c* and *d*). This may be because, as mentioned, p57^Y63^ does not bind in the same position as p27^Y74^ in the trimer (Fig. 2). Therefore, this could explain why phosphorylation of p57^Y63^ has less of an effect on the structure of the CyclinA/Cdk2 complex than phosphorylation of p27^Y74^.

**Figure 5:**
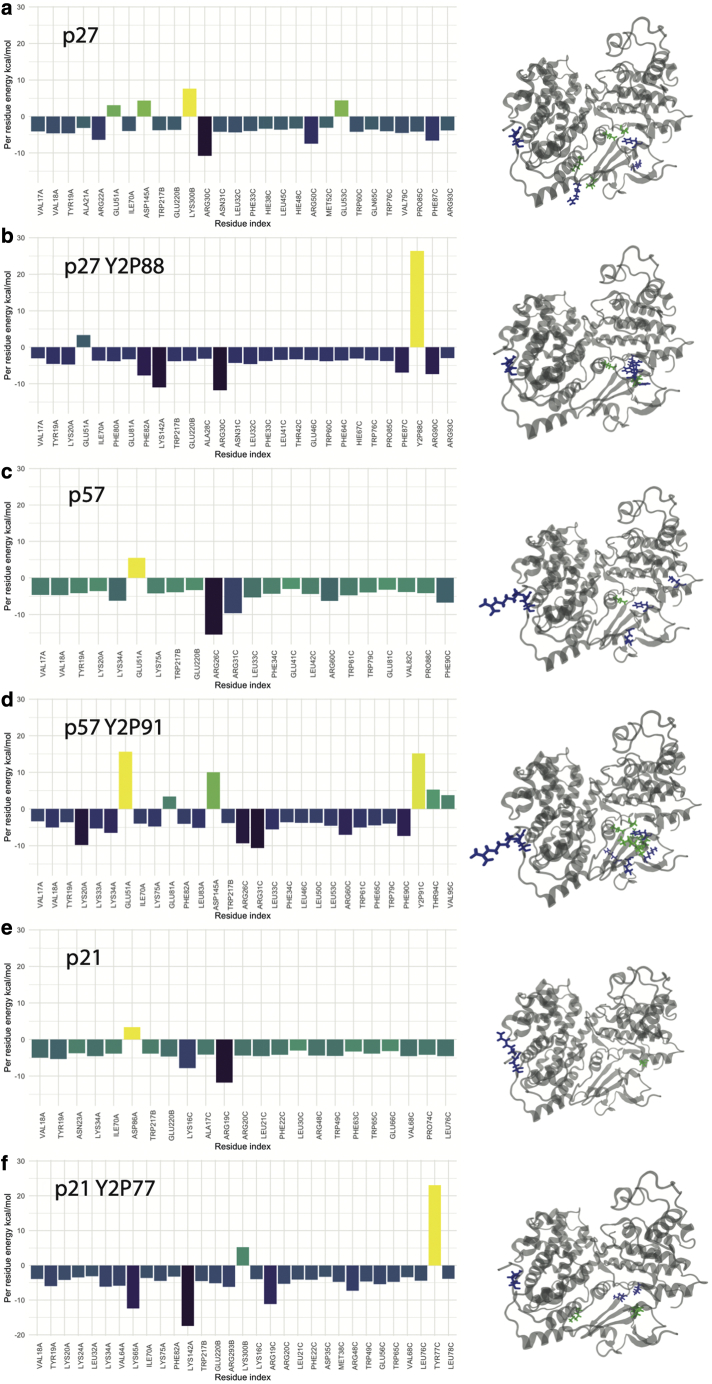
Per-residue molecular mechanics Poisson-Boltzmann surface area energy decomposition averaged over the last 400 ns of simulation. Cdk2 is chain A, Cyclin is chain B, and pX is chain C. Residues that have a positive (destabilizing) contribution to the binding free energy are shown on the structure in green, and the negative (stabilizing) contribution is shown in blue. Only per-residue contributions above +/−2 kcal/mol are represented on the molecular mechanics Poisson-Boltzmann surface area plots for clarity. Per-residue contributions below these values do not significantly contribute to binding. To see this figure in color, go online.

**Figure 6:**
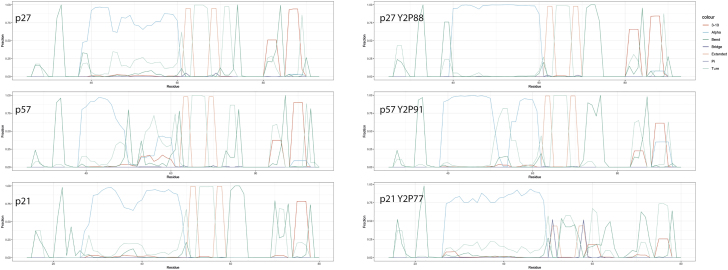
The secondary structure of p27, p57, and p21 and their phosphorylated form identified using the DSSP method (44) from the last 400 ns of each simulation. To see this figure in color, go online.

Unlike the hydrogen-bonding-network analysis that is based on donor-acceptor distances and angles, the Gibbs free energy of binding is calculated from the force-field energies. The energies of the solvated ligand and receptor are subtracted from the complex to leave the free energy of binding. The energy decomposition in Fig. 5 displays the contribution of each residue in the ternary complex to the overall binding affinity. Here, we only consider those tyrosine residues universally conserved with p27^Y88^ to analyze the effect of their phosphorylation on potential 3_10_ helix ejection from the active site and CDK2 activation. For the CyclinA/Cdk2/p27 complex, the residues that bind most strongly are p27^R30,R50^, and the residue that destabilizes the binding is CyclinA^K300^. When p27^Y88^ is phosphorylated, shown in Fig. 5 *b*, we see a change in the pre-residue contributions to the binding affinity and their corresponding magnitudes. p27^R30^ continues to bind tightly, but p27^Y2P88^ becomes the most destabilizing residue with an energy of 26 kcal/mol. Since Y88 is a key residue in the $3_{10}$ helix, this gives us a clear insight into why we see ejection of the p27 helix after Y88 phosphorylation. When p57 is phosphorylated, we see a large increase in the binding affinity at residue p57^Y2P91^, but interestingly, we see an increase in energy in Cdk2 at residues Cdk2^E51,E81,D145^ that was not observed when phosphorylating p27^Y88^ and could explain the increased rate of dissociation of the helix for phosphorylated p57 (Fig. 4). In addition, the increased free-energy contribution of p57^T94,V95^ upon p57 phosphorylation may also contribute to the rapid dissociation of the $3_{10}$ helix in p57, as no similar increase is seen in near-adjacent residues in p27 and p21 after their phosphorylation. If p21 is phosphorylated at residue Y77, we see a jump of 23 kcal/mol at residue p21^Y2P77^, similar to the magnitude of the change seen upon p27 phosphorylation. However, unlike what is observed in p27, after p21 phosphorylation, we see a large decrease in the pre-residue free energy of Cdk2^K65,K142^ to between 10 and 20 kcal/mol that acts to offset the free-energy increase in p21^Y2P77^, leading to a stable ternary complex and no ejection of the $3_{10}$ helix in the p21 model, in the timescales simulated here. The secondary structures of p27, p57, and p21 are shown in Fig. 6. The overarching trend in structural motifs for these inhibitors is that the alpha-helix character around residues 40–60 is increased when each is phosphorylated. This could change the accessibility of residues in this region (i.e., p27^Y74^ and p57^Y61^) for phosphorylation when p27^Y88^ or p57^Y91^ are phosphorylated. There is also a trend in that the $3_{10}$ helical character is reduced, particularly with p57^Y2P91^ and p21^Y2P77^.

To more easily and directly compare the structural effects of phosphorylation of the conserved p27^Y88^, p21^Y77^, and p57^Y91^ residues, we generated animations of the unphosphorylated and phosphorylated forms (Video S1). Helix ejection and reduction in the $3_{10}$ helical character of phosphorylated p57^Y91^ is clearly visible at these timescales. Loss of $3_{10}$ helical character is also observed in p21 phosphorylated at Y77, yet p21 still blocks the Cdk2 active site. We also observed that in phosphorylated p27^Y88^ and p57^Y91^, a cooperative change appears to occur upon tyrosine phosphorylation where the C lobe of Cdk2 becomes more mobile (Video S1). This does not occur in phosphorylated p21^Y77^. The significance of this is, at present, uncertain. However, it is worth noting that the T-loop of Cdk2 lies just beneath the C lobe. Since p21 and p27 are thought to block CAK-mediated phosphorylation of T160 in Cdk2 (51), it is tempting to speculate that increased mobility of the C-lobe of Cdk2 could play a role in permitting CAK-mediated activation of Cdk2, a model observation that could be experimentally tested in the future with in vitro kinase assays.

Video S1. The molecular dynamics trajectories from CyclinA:CDK:p27 (PDB: 1JSU) and homology models of CyclinA:CDK in complex with p21 and p57 are shown in the top row, along with the conserved tyrosine phosphorylated inhibitor complexes (p27-Y88p/p21-Y77p/p57-Y91p) in the bottom row The intramolecular peptide backbone RMSD is shown highlighting the ejection of the 310 helix in tyrosine phosphorylated p57 in cMD simulations. Also note the increased mobility of the C-lobe of CDK2 (at the top of the shown structures) in tyrosine-phosphorylated p27 and p57 complexes, but not in phosphorylated p21 trimers.

In summary, although all Cip/Kip proteins share conserved tyrosine residues, our cMD simulations suggest that phosphorylation of these residues has different effects on CyclinA/Cdk2/CKI complexes. While the individual effects are described above, this is also significant as it means that we should remain cautious when extrapolating changes that have been observed to occur in p27 and bound CyclinA/Cdk2 complexes after p27 tyrosine phosphorylation to the other conserved Cip/Kips.

### Hierarchy and cooperativity of tyrosine phosphorylation in Cip/Kips

A question that arises when considering the phosphorylation of protein residues is whether the amino acid is exposed and accessible to kinases. While solvent exposure alone is not the only criterion for a residue to be phosphorylated, it is reasonable to assume that the more exposed a residue is, the greater is its likelihood of being phosphorylated by specific kinases. Many residues are not solvent exposed in the unphosphorylated state, which begs the question of how such residues are modified (29). Although the main consideration of this study is to understand the structural and dynamical effects of phosphorylation on these complexes once phosphorylated, it is important to consider the potential of these inhibitors to be modified via phosphorylation. This is particularly relevant here as it has been shown that Y88 in p27 is buried against the surface of Cdk2 (Fig. S5) yet can still be phosphorylated by NRTKs due to intrinsic flexibility in p27 (30). However, one would predict that more solvent-exposed residues would have an increased probability of being phosphorylated and may induce conformational changes to promote phosphorylation of other residues, potentially imposing a hierarchy in Cip/Kip phosphorylation (29).

To determine how solvent exposed each tyrosine phosphorylation site is in all three Cip/Kips, in the presence and absence of other phosphorylated tyrosines, we calculated the solvent-accessible surface area (SASA) using the linear combinations of pairwise overlaps algorithm (52). From our sequence alignment analysis in Fig. S1, we have shown the potential importance of several tyrosine residues in terms of their conservation. Previous experimental studies have shown the potential for these tyrosines to be phosphorylated, either in cells or in vitro. (25,53,54).

**Figure 7:**
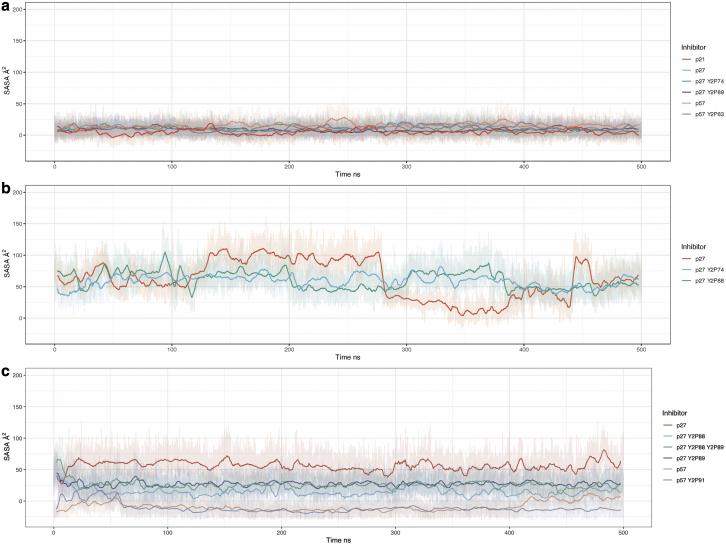
Solvent-accessible surface area of three tyrosine residues of interest: (*a*) tyrosine Y88 in p27, which is positioned within the active-site pocket, and the equivalent tyrosine position in p57 and p21 (position Y91 and Y77 respectively); (*b*) p27 position Y89, positioned within the Cdk2 active site pocket but only present in p27; and (*c*) distal from the active site, p27 Y74, and near-equivalent site Y63 in p57. To see this figure in color, go online.

We first considered the tyrosine residues that sit in the Cdk2 active site. Fig. 7 *a* shows the potential for Y88 in p27, and the corresponding positions in p21 and p57, to be phosphorylated in the presence and absence of phosphorylation of other tyrosine residues (listed on the right-hand side). The SASA of tyrosine at this position (p27^Y88^, p21^Y77^, p57^Y91^) remains close to zero in all models, telling us that throughout these cMD simulations, these residues remain solvent inaccessible and buried and in this state are unlikely to be phosphorylated. Fig. 7 *b* shows the SASA of Y89, which is only present in p27. Unlike its neighbor Y88, Y89 fluctuates between accessible and inaccessible states in otherwise non-tyrosine phosphorylated p27 (Fig. 7 *b*, *red*). When Y74 or Y88 are phosphorylated, Y89 remains in a solvent-accessible state (see around 280–400 ns in Fig. 7 *b*), suggesting cooperativity between these phosphorylation events.

p27^Y74^ remains solvent accessible in its canonical, unmodified, form, whereas p57^Y63^ appears to be buried and inaccessible. Fig. S5 demonstrates the difference between the accessibility of these two residues, once again highlighting that while both tyrosines can be phosphorylated by NRTKs (at least in vitro in the case of p57 (25)), they do not appear to be equivalent in terms of function.

To quantify the potential for tyrosine phosphorylation of these residues, we calculated the density of SASA taken from three independent cMD simulations (Fig. S6). This confirms what we see for residues p27^Y88^, p57^Y91^, and p21^Y77^—that these residues are buried and inaccessible to solvent, which would imply that they are not available for modification by phosphorylation under these conditions and on these timescales (Fig. S6 *a*). p27^Y89^ resides in an accessible state in our simulations (Fig. S6 *b*).

Together, these data suggest that the tyrosine residues that sit in the Cdk2 active site (p27^Y88^, p21^Y77^, and p57^Y91^) are inaccessible to phosphorylation. The exception to this is p27^Y89^, which remains accessible. p27 is the only Cip/Kip to have a double YY motif in this position. Since experimental data have shown that these sites can be phosphorylated, our simulations indicate that phosphorylation must occur under modifying conditions. For p27 Y88, that could be brief exposure “dynamic anticipation” of the residue (30). Similar mechanisms could be at play for p21 and p57, or perhaps NRTK binding alters residue exposure.

### A predicted route to Cdk2 activation by Cip/Kip release

**Figure 8:**
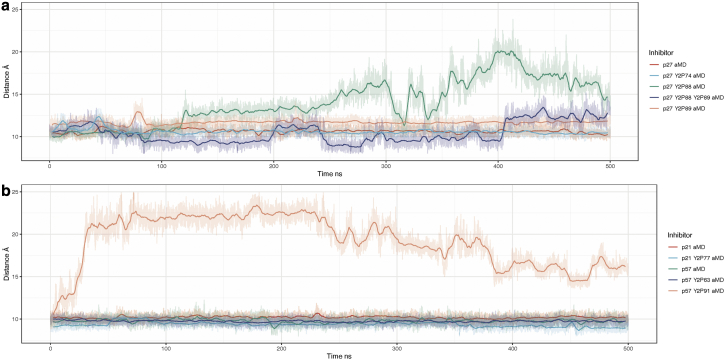
Center-of-mass distance between the $3_{10}$ α helices of (*a*) p27, p27 Y2P74, p27 Y2P88, p27 Y2P88 Y2P89, p27 Y2P89 and (*b*) p21, p21 Y2P77, p57, p57 Y2P63, p57 Y2P91 and the active-site pocket. Some equilibrium distances appear to be above/below 10 Å because as the phosphorylation state is altered, the center of mass of that group of atoms will be shifted. Of all the various tyrosine phosphorylation states of p27, we only observe dissociation of the inhibitory $3_{10}$ α helix when Y88 has been phosphorylated. When both Y88 and Y89 are phosphorylated, the α helix fluctuates between a bound and partially unbound state, indicating that phosphorylated Y89 might aid in preventing the dissociation of the inhibitor when Y88 is phosphorylated. To see this figure in color, go online.

CyclinA/Cdk2 regains partial activity by ejecting the p27 $3_{10}$ helix from the active site (30,31). This event precedes intramolecular phosphorylation of p27 at T187 that targets p27 for proteasomal degradation and would lead to “full” activation of Cdk2 (17). The $3_{10}$ helix is present in each of the Cip/Kip inhibitors (Fig. 2). To assess the potential for Cdk2 activation in each complex, we monitored the distance between the center of mass of the inhibitor’s $3_{10}$ helix and the active-site pocket. A greater distance suggests a more active kinase, in that the active-site pocket is no longer obstructed. Here, we used aMD simulations since it has been previously shown that the $3_{10}$ helix ejection in p27 happens on slow timescales and is not visible in cMD simulations (30). These distances are shown as a time series for each phosphorylation state in Fig. 8 from aMD simulations.

**Figure 9:**
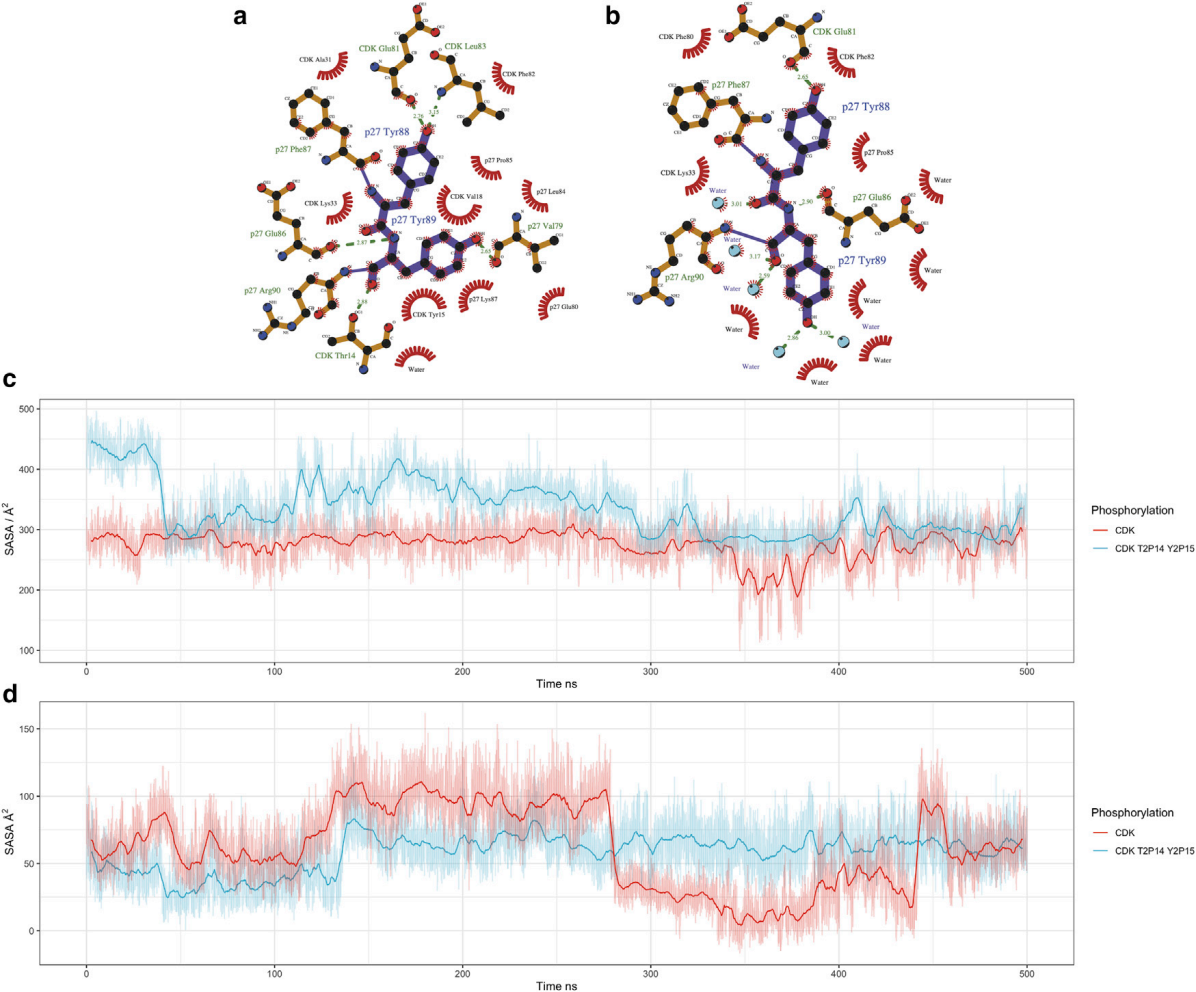
(*a*) Two-dimensional rendering of residues Y88 and Y89 in p27 taken from the centroid structure of two k-means clusters. (*b*) Distance between p27 residue Y89 and the center of mass of Cdk2 residues T14 and Y15. (*c*) The solvent-accessible surface area of Cdk2 T14 and Y15. (d) The solvent-accessible surface area of p27 Y89 when Cdk2 residues T14 and Y15 are phosphorylated to T2P14 and Y2P15. To see this figure in color, go online.

Our models suggest that the only circumstances in which the $3_{10}$ helix blocking the ATP-binding site is expelled is in models where the buried tyrosine residue is phosphorylated, i.e., positions Y88 in p27 and Y91 in p57. Double phosphorylation of Y88 and Y89 in p27 does not lead to the expulsion of the helix, suggesting that phosphorylation of these adjacent residues have different structural effects (Fig. 8). This prediction that Y89 phosphorylation compensates for the destabilizing phosphorylation of Y88 in that it acts to stabilize the $3_{10}$ helix in the Cdk2 active site when Y88 is also phosphorylated can be explained by the hydrophilic nature of phosphorylated tyrosine. Specifically, unphosphorylated tyrosine is slightly hydrophobic, and, as such, it is energetically favourable for Y88 and Y89 to be buried in the CDK2 active site, shielded from bulk solvent. Tyrosine phosphorylation makes tyrosine polar and extremely hydrophilic. This energetic environmental change will drive p27^Y2P88^ from the hydrophobic pocket into the solvent, ejecting the $3_{10}$ helix. However, Y89 is already partially exposed to the solvent (Fig. 9 *c*), and so ejection of the $3_{10}$ helix is not required to expose Y89 to the solvent in order to alleviate the change in energy when Y89 is phosphorylated and hydrophilic. Therefore, phosphorylated Y89 could stabilize the bound state when Y88 is also phosphorylated.

The rate of $3_{10}$ helix dissociation is greater for p57^Y91^ phosphorylation (Fig. S7) than for p27^Y88^ phosphorylation. In fact, in p57, when Y91 is phosphorylated, the helix can dissociate during cMD simulations over 500 ns (Fig. S8; Video S1). Experimental data show that, despite being buried, p57^Y91^ can be phosphorylated by Abl kinase, at least in vitro (25). Therefore, our simulations suggest that while there is potentially a low probability of p57 Y91 phosphorylation, in the absence of other modifying factors, once Y91 is phosphorylated, Cdk2 would be rapidly activated (Fig. 8).

For the case of p21 when the similarly positioned tyrosine Y77 is phosphorylated, we do not observe any dissociation of the helix from the Cdk2 active site, even in aMD simulations (Fig. 8). This leads us to believe that there is no reactivation of Cdk2 over these time scales when Y77 in p21 is phosphorylated as the active site remains obstructed. Also, our previous analyses on the RMSD of p21^Y77^ phosphorylated complexes predicted increased hydrogen bonding within the trimer, suggesting that p21 would remain a bound inhibitor.

Together, our simulations suggest that the sensitivity to the ejection of the $3_{10}$ helix from the Cdk2 active site differs between the three Cip/Kip inhibitors, with p57 being the most sensitive to release after tyrosine phosphorylation and p21 not being released at all. Moreover, even though Y88 and Y89 are adjacent in p27, their phosphorylation does not have the same effect.

### Inhibition of Cdk2 by Cip/Kips and Wee1 are mutually exclusive

Our models and simulations reveal that p27 binding to CyclinA/Cdk2 blocks access to the inhibitory phosphorylation sites T14 and Y15 in the Cdk2 active-site pocket (Fig. 9 *a*). These models, together with published experimental data (55), suggest that the p27 has to be removed from CyclinA/Cdk2 before Myt1 and Wee1 can phosphorylate Cdk2. Similarly, if we simulate the phosphorylation of T14 and Y15 in the CyclinA/Cdk2/p27 trimer, the N-terminus of Cdk2 becomes more diffuse, suggesting that it is no longer bound to p27 (Fig. 9; visualized in Fig. S9).

In Cdk2 that is unphosphorylated at T14 and Y15, the N-terminal domain of Cdk2 is not free and exposed. The red time series in Fig. 9 *c* shows the SASA of p27 Y89. The two states visible, either accessible or not accessible to water, arise due to intermolecular hydrogen bonds between Cdk2 T14 and p27 Y89 that have a lifetime of a couple of hundred nanoseconds. This is shown (Fig. 9 *a*) as a two-dimensional LigPlot (56) of residue positions surrounding p27^Y88,Y89^ taken from the centroid structure from the two conformational ensembles mentioned. When Cdk2 is phosphorylated at residue T14 Y15, we see this open/close conformational switch removed, and the N-terminal domain of Cdk2 becomes unbound to p27 and diffuse (Fig. 9 *c*). Together, these data suggest that inhibition of Cdk2 by Cip/Kips and inhibitory kinases are mutually exclusive events, which has implications for Cdk2 activation during S-phase entry.

### Comparison of Cip/Kip binding and release between Cdk2 and Cdk4

**Figure 10:**
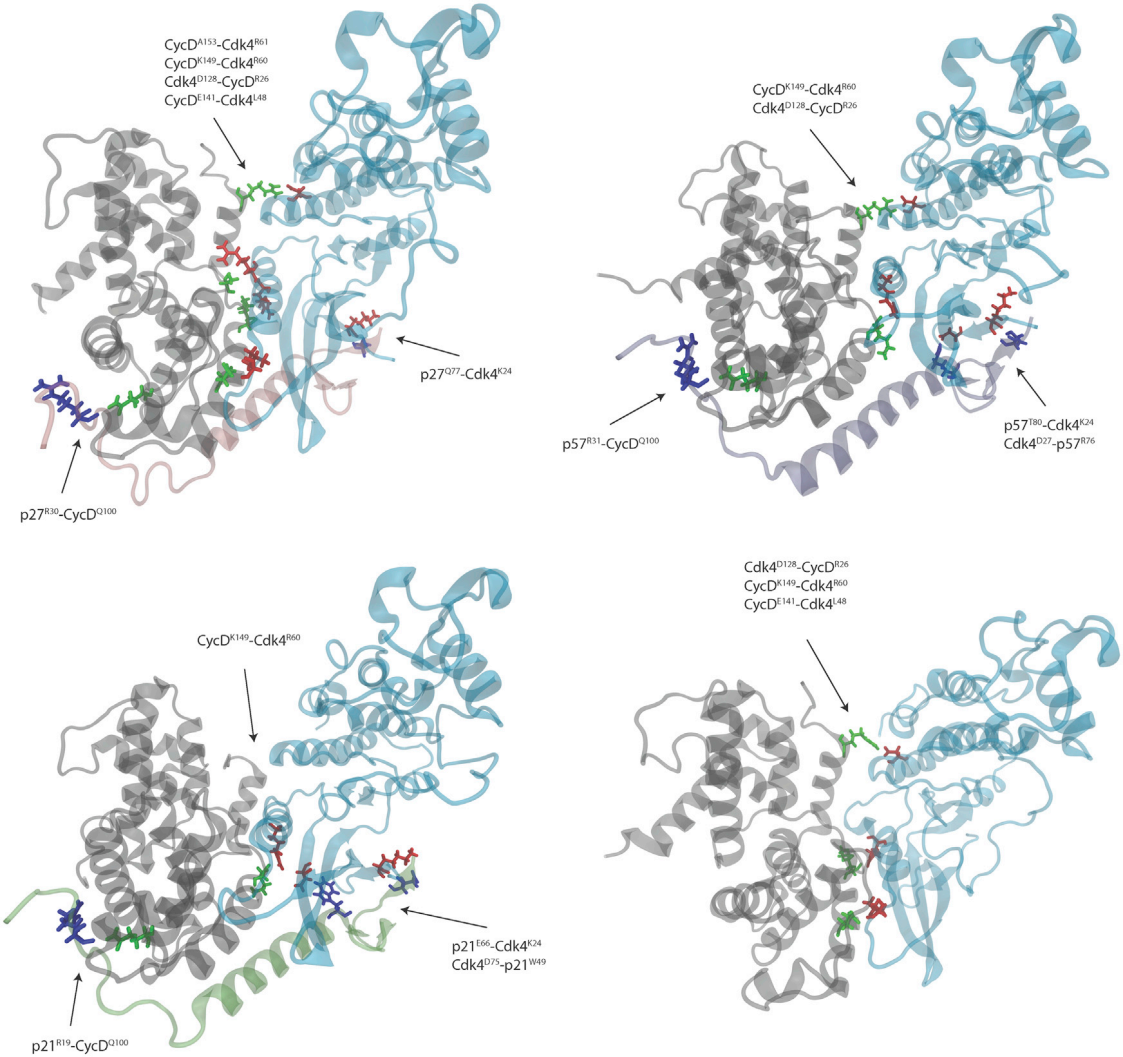
Structures taken from the final frame of each cMD simulation at 500 ns. Intermolecular hydrogen bonds were calculated over the last 400 ns of simulation, and contacts between molecules with a fraction lifetime above 0.5 are displayed on the full structure in the order of hydrogen bond acceptor first and donor second. This simplifies and shows the major, long-lived intermolecular contacts between Cdk4 and CyclinD and their inhibitors. Residues are colored according to the molecule they belong to: blue is CKI, green is CyclinD, and red is Cdk4. To see this figure in color, go online.

The recently solved structures of CyclinD/Cdk4/p27 and CyclinD/Cdk4/p21 allow us to compare Cdk2 and Cdk4 regulation by the Cip/Kip proteins (13). We constructed models to compare the binding of all three Cip/Kips to CyclinD/Cdk4 (see Fig. S10 for a side-by-side comparison of Cdk2and Cdk4 structures). Analysis of RMSD shows CyclinD/Cdk4/CKI models equilibrating after 25 ns of initial simulation, like those we reported for CyclinA/Cdk2/CKI (Fig. S11). We calculated all intermolecular hydrogen bonds between CyclinD/Cdk4/CKI and observed three main domains of interaction, analogous to those seen in the Cdk2 ternary complex (Fig. 10; Tables S6–S9). These are 1) between Cdk4 and CyclinD via the Cdk2 C-helix, 2) between CKI and Cdk4, and 3) between CKI and CyclinD. Where p27^Y88^, p21^Y77^, and p57^Y91^ binding is the dominant interaction in the Cdk2 models, representing interactions between the $3_{10}$ helix and the Cdk2 active site, the most significant interacting residues in the Cdk4 models are p27^Q77^, p57^T80^, and p21^E66^ bound to Cdk4^R24^. This may explain why no ordered structure for this helical region was observed in the electron density map for the CyclinD/Cdk4/CKI structure (13).

Our analysis of interactions over the course of the cMD simulations reveal differences between the three CKIs when bound to the dimer. p21 and p57 have an interaction close to the Cdk4 active site (p21^W49^ and p57^R76^), which is not observed in p27. It appears that this additional interaction leads to a change in the hydrogen bonding between CyclinD/Cdk4—effectively pulling apart the Cdk4^L48^-CyclinD^E141^ interaction (Fig. 10). This suggests that p27 would act as a better assembly factor for CyclinD/Cdk4 complexes than p21 or p57.

**Figure 11:**
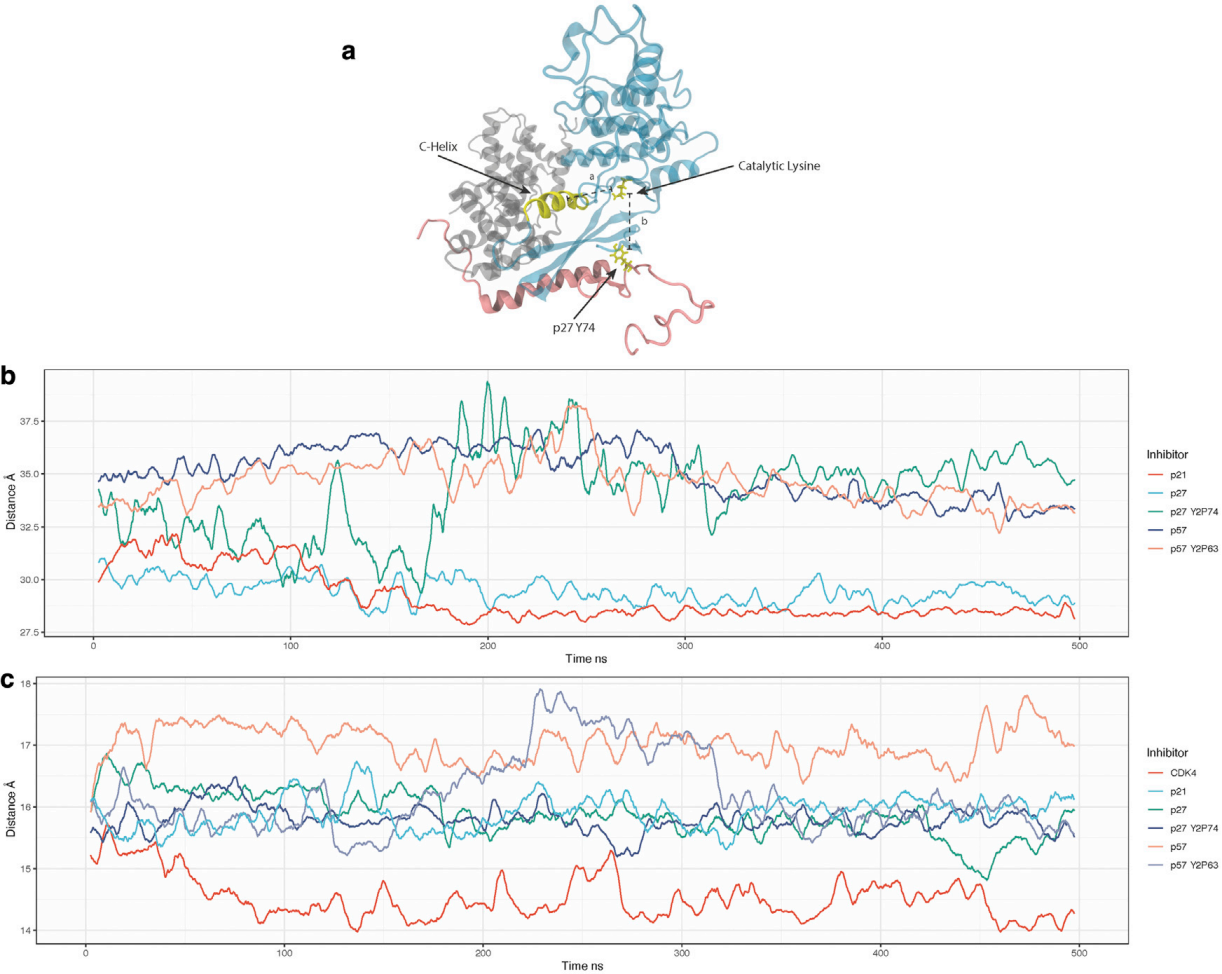
(*a*) Key structural features of CyclinD/Cdk4/p27 complex. (*b*) The distance between amino-acid residue 74 of the three Cip/Kip inhibitors in different states of phosphorylation, and the center of mass of Cdk4. As the dislocation of residue 74 from the complex is known to lead to activation of Cdk4, we show that this as an indicator of potential activity. (*c*) The distance between the catalytic lysine in Cdk4 and the center of mass of the Cdk4 C helix. To see this figure in color, go online.

Unlike CyclinA/Cdk2, which is active in the dimeric complex, CyclinD binding alone does not induce catalytic activity in Cdk4 (13). Compared with CyclinA/Cdk2, where Cip/Kips inhibit kinase activity, Cip/Kip binding can both inhibit and promote CyclinD/Cdk4 activity. Therefore, we cannot infer Cdk4 activity from the absence of a Cip/Kip blocking the active site alone—such as the $3_{10}$ helix in the case Cdk2 inhibition. In Fig. 11 *a*, we combine two distances to infer the potential for Cdk4 activity. With both distances, the closer the catalytic lysine is to either the C helix or the p27^Y74^ (or equivalent) residue, the less active Cdk4 is.

Fig. 11*b* shows the distance between p27^Y74^, the corresponding amino acid in p21 (which is not a tyrosine), p57^Y63^, and the center of mass of Cdk4 (Fig. 11 *a*, *distance b*). This distance tells us whether the tyrosine residue is tightly bound to the Cdk4 molecule, suggesting that the Cdk4 molecule is inactive. Of the models studied, unphosphorylated p21 and p27 are tightly bound at this position, suggesting that Cdk4 is inactive (Fig. 11 *b*). If Y74 is phosphorylated in p27 (green line), Y2P74 transitions from bound to unbound at around 150 ns, and we predict that Cdk4 would become more active—consistent with published results (13). p57, in both unphosphorylated and Y63-phosphorylated forms, is unbound at this position throughout the simulation, which suggests that CyclinD/Cdk4 is further from its dimeric form when p57 is bound, making p57 a less potent inhibitor. We compared the position of p57^Y63^ in the CyclinD/Cdk4 structure with that of Y74 in p27 and observed a different orientation in binding relative to the Cdk4 beta sheet where Y63 points away from the dimer interface (Fig. S12). This explains why this region is unbound in our simulations.

A second measure of the potential for CyclinD/Cdk4 to be active is the distance between the Cdk4 C helix and the catalytic lysine residing in the active site (Fig. 11 *b*). This is a measure of the “openness” of the active site and a proxy for Cdk4’s ability to accept ATP. Without one of the Cip/Kip family of inhibitors, CyclinD/Cdk4 (Fig. 11 *c*, *red*) exhibits the smallest distance between the C helix and the catalytic lysine over the course of a 500 ns simulation, suggesting that Cdk4 is inactive. Considering both of these distances together, that is, the distance between the catalytic lysine and C helix and between p27^Y74^ and Cdk4 (shown in Fig. 11 *a*), allows us to predict Cdk4 activity in these inhibitor-bound states. Taking both measurements into account, we suggest the following order of activity for CyclinD/Cdk4-bound complexes: p21 (inactive) = p27 (inactive) and p27 Y2P74 (partially active) < p57 Y2P63 (active) < p57 (active).

Our MD simulations are consistent with published experimental data (13) showing how phosphorylation of Y74 in p27 bound to CyclinD/Cdk4 can promote activity by inducing conformational changes in the CyclinD/Cdk4 complex. Furthermore, our analyses of p57 bound to CyclinD/Cdk4 suggest that p57 may be a worse inhibitor of CyclinD/Cdk4 activity than of CyclinA/Cdk2 activity, which is consistent with published data (57,58).

## Discussion

How mitogenic signaling can regulate context-dependent cell proliferation is not fully understood. Here, we have tackled this problem by investigating how mitogens, via the activation of tyrosine kinases, regulate structural changes in Cyclin/Cdk/CKI trimers that ultimately drive Cdk activation and cell proliferation. Our findings reveal that despite shared structural features, tyrosine phosphorylation of CKIs promotes non-conserved and non-intuitive changes in Cyclin/Cdk/CKI complexes and, therefore, Cdk activity. Our models have generated a number of predictions, and below, we discuss the significance of these predictions and how they could be experimentally tested in the future.

Cip/Kip inhibitors are intrinsically disordered proteins that fold upon binding to Cyclin/Cdk dimers. Our simulations show that notable differences exist in the strength of hydrogen bonds between Cyclin/Cdk/CKI proteins. For example, p21 binding to Cyclin/Cdk appears to destabilize interactions between CyclinA/Cdk2 and CyclinD/Cdk4, which may be an additional mechanism of Cdk inhibition by p21. p21 also has an additional strong hydrogen bond to Cdk2, with N23 in the Cdk2 active site, that is not present in p27 or p57. This, together with our observations that phosphorylation of p21^Y77^ in the Cdk2 active site does not promote ejection of the $3_{10}$ helix of p21, suggests that, under mitogenic signaling, p21 inhibits Cdk2. Together with the fact that p21 also lacks the equivalent p27^Y74^ residue and so remains a Cdk4 inhibitor on mitogen stimulation, our analyses could explain how p21 is able to maintain cell-cycle arrest in the presence of DNA damage and ongoing mitogenic signaling (26,59). Experimental data from NMR spectroscopy suggests that the $3_{10}$ helix of p21 is released from the Cdk2 active site on Y77 phosphorylation but that p21 remains a bound inhibitor (25). Our analyses suggest a loss of the $3_{10}$ helical structure upon p21^Y77^ phosphorylation but not its ejection. One explanation for the difference between our simulations and experimental measurements could be that the tyrosine kinase (Abl in this case) induces a conformational change in p21 that promotes helix ejection. These model predictions can be experimentally tested, for example, by solving the crystal structures of p21 and p57 bound to CyclinA/Cdk2 and of CyclinA/Cdk2/p21 in the absence or presence of Abl kinase.

Our SASA measurements demonstrate that, with the exception of p27^Y89^, all Cip/Kip tyrosine residues that sit in the Cdk2 active site are inaccessible to phosphorylation. Yet, these sites can be phosphorylated. For p27, previous work demonstrated that p27 exhibits intrinsic flexibility, such that p27 can briefly exist in states where Y88 is exposed for sufficient time to be phosphorylated (30). Similar flexibility may exist in p21 and p57. Alternatively, kinase binding may induce conformational changes that promote tyrosine exposure. A third mechanism could be that phosphorylation of other tyrosine sites induces conformational changes that promote exposure of residues on the $3_{10}$ helix. However, we found no evidence for p27^Y74^ or p57^Y63^ phosphorylation increasing solvent accessibility of p27^Y88^ or p57^Y91^. Conversely, it was recently reported that p27^Y88^ phosphorylation increased the exposure of p27^Y74 29^, which we did not observe in our simulations. In the models in (29), p27^Y89^ was mutated to p27^Y89F^ to mimic previous experimental conditions, and therefore this may have affected how p27 behaved in the simulations. Therefore, we favor a combination of intrinsic flexibility and kinase-mediated conformational changes promoting tyrosine exposure.

p27 is the only Cip/Kip to have two adjacent tyrosine residues, Y88 and Y89, in the $3_{10}$ helix. Despite being neighbors, the two residues behave differently. While Y88 is buried against Cdk2 and solvent inaccessible, Y89 fluctuates between accessible and inaccessible states, making it more exposed for phosphorylation. However, despite an increased likelihood of phosphorylation, Y89 phosphorylation does not initiate $3_{10}$ helix ejection under the conditions used in our simulations. In fact, simultaneous phosphorylation of Y89 with Y88 appears to suppress helix ejection and Cdk2 activation. This is intriguing and could be linked to preventing Cdk2 activation and cell proliferation under conditions of high levels of tyrosine kinase signaling, for example, as observed upon oncogenic mutation. Previous work investigating the accessibility of Y88 in p27 did not investigate the accessibility of Y89 (30). However, similar single-molecule multiparameter fluorescence detection experiments, as performed in that work for p27^Y74^ and p27^Y88^ to investigate p27^Y89^ accessibility and flexibility, would determine if our model prediction is correct.

Our simulations of Cip/Kip inhibitors bound to CyclinD/Cdk4 were able to recapitulate recently reported features of Cdk4 activation and inhibition by p27 and p21 (13). We were also able to show that p57 binding does not promote as extensive hydrogen bonding between CyclinD and Cdk4 as p27 does, suggesting that p57 may not promote CyclinD/Cdk4 complex assembly in the same way as p27, another prediction that could be addressed by solving the structure of CyclinD/Cdk4/p57. Our analyses also suggest that p57 may be a poor inhibitor of Cdk4 activity, since in the presence of Y63-phosphorylated or unphosphorylated p57, the distance between the catalytic lysine and the C helix or p57 is large, suggesting a potentially active Cdk4. Our models suggest that ejection of the $3_{10}$ helix of p57 from the Cdk2 active site is very sensitive to phosphorylation of p57^Y91^, a prediction that is experimentally testable using biophysical methods such as NMR. p57 is expressed at high levels during embryogenesis, and its expression decreases, becoming much more restricted at embryonic day 13.5 and only being expressed in a limited number of adult tissues (60). This is unlike p21 and p27, which are more ubiquitously expressed. Together with our data suggesting that p57 may be a poor inhibitor of CyclinD/Cdk4, this suggests that in tissues where both p27 and p57 are expressed, phosphorylation of p27 would be the rate-limiting factor in promoting cell-cycle entry.

Recent experimental data have suggested that Cdk4/6 inhibitors being used in the clinic in the treatment of Her2-negative/estrogen-receptor-positive breast cancer, for example, Palbociclib, may promote cell-cycle arrest by forcing the redistribution of Cip/Kip inhibitors from Cdk4 onto Cdk2 (13). This uncertainty over the mechanism of action of Cdk4/6 inhibitors stresses the need for an in-depth, mechanistic understanding of how Cdk activity is controlled by Cip/Kip inhibitors, in order to improve patient stratification and motivate the development of improved CKIs. Our analyses go some way toward achieving a molecular understanding of Cdk activation required for cell proliferation and provide a basis for further experimental exploration in this area.

## Author contributions

Conceptualization, J.B.S. and A.R.B.; methodology, J.B.S., K.L.M., and T.W.; investigation, J.B.S.; visualization, J.B.S. and K.L.M.; supervision, A.R.B. and T.W.; writing, J.B.S. and A.R.B.

## Supporting material

Document S1. Tables S1–S9, and Figures S1–S12

Data S1. Homology model of CyclinA/Cdk2/p21

Data S2. Homology model of CyclinA/Cdk2/p57

Document S2. Article plus supporting material
