# Supplementary material for: Conserved Cdk inhibitors show unique structural responses to tyrosine phosphorylation
Source: Biophys J. 2022 May 25;121(12):2312–29. doi: 10.1016/j.bpj.2022.05.024 (PMC9279356; doi:10.1016/j.bpj.2022.05.024)
Supplement: Document S1. Tables S1–S9, and Figures S1–S12 [file mmc1.pdf]

Biophysical Journal, Volume *121*

Supplemental information

Conserved Cdk inhibitors show unique structural responses to tyrosine phosphorylation

Jacob B. Swadling, Tobias Warnecke, Kyle L. Morris, and Alexis R. Barr

Conserved Cdk inhibitors show unique structural responses to tyrosine phosphorylation

Jacob B. Swadling,[*,†,‡] Tobias Warnecke,[†,‡] Kyle L. Morris,[‡] and Alexis R. Barr[*,†,‡]

†*Institute of Clinical Sciences, Imperial College London, UK*

‡*MRC London Institute of Medical Sciences, UK*

E-mail: jacob.swadling@lms.mrc.ac.uk; a.barr@lms.mrc.ac.uk

Supplementary Information

Accelerated molecular dynamics

aMD can capture high energy transition states by modifying the potential energy surface through the addition of a non-negative boost potential $\Delta V(r)$ to the original potential $V(r)$, whenever $V(r)$ is below a pre-defined energy level. The modified potential is related to the true potential, bias potential and boost energy by:

$$V^{*}(r) = \begin{cases} V(r) & V(r) \geq E, \\ V(r) + \Delta V(r) & V(r) < E, \end{cases} \tag{1}$$

The choice of $\Delta V(r)$ is given by:

$$\Delta V(r) = \frac{(E - V(r)^2)}{\alpha + (E - V(r))} \tag{2}$$

where α is a tuning parameter that determines how deep the modified potential energy basin is. E and α were selected using the following equations:

$$\begin{aligned} E_{dihed} &= V_{dihed\_avg} + 3.5N_{res},\ \ \alpha_{dihed} = 3.5N_{res}/5 \\ E_{total} &= V_{total\_avg} + 0.175N_{atoms},\ \ \alpha_{total} = 0.175N_{atoms} \end{aligned} \tag{3}$$

The correct canonical ensemble average of the system was obtained by re-weighting using cumulant expansion to the second order which has been demonstrated to recover the most accurate free energy profiles within statistical errors of $\sim k_BT$, particularly when the distribution of the boost potential exhibits low anharmonicity (*i.e.*, near-Gaussian distribution), such as the case here.[1] The Canonical ensemble distribution $p(A)$, can be recovered using:

$$p(A_j) = p^*(A_j)\frac{\langle e^{\beta\Delta V(r)}\rangle_j}{\sum_{j=1}^{M}\langle e^{\beta\Delta V(r)}\rangle_j},\ j = 1, ..., M \tag{4}$$

where M is the number of bins, $\beta = 1/k_BT$ and $\langle e^{\beta\Delta V(r)}\rangle_j$ is the ensemble averaged Boltzmann factor of $\Delta V(r)$ for simulation frames found in the jth bin. The ensemble-averaged reweighting factor can be approximated using cumulant expansion to the second order:[1]

$$\begin{aligned} \langle e^{\beta\Delta V(r)}\rangle &= exp\left\{\sum_{k=1}^{\infty}\frac{\beta^k}{k!}C_k\right\} \\ C_2 &= \langle\Delta V^2\rangle - \langle\Delta V\rangle^2 = \sigma_{\Delta V}^2 \end{aligned} \tag{5}$$

Supplementary Table 1: Simulation Details

Model	Inhibitor	Cdk	Cyclin	cMD	aMD
1	none	Cdk2	CyclinA	500 ns	500 ns
2	p27	Cdk2 T2P13	CyclinA	500 ns	500 ns
3	p27	Cdk2 Y2P14	CyclinA	500 ns	500 ns
4	p27	Cdk2 T2P13/Y2P14	CyclinA	500 ns	500 ns
5	p27	Cdk2	CyclinA	500 ns x3	500 ns
6	p27 Y2P88	Cdk2	CyclinA	500 ns x3	500 ns
7	p27 Y2P89	Cdk2	CyclinA	500 ns x3	500 ns
8	p27 Y2P88 Y2P89	Cdk2	CyclinA	500 ns x3	500 ns
9	p27 Y2P74	Cdk2	CyclinA	500 ns x3	500 ns
10	p21	Cdk2	CyclinA	500 ns x3	500 ns
11	p21 Y2P77	Cdk2	CyclinA	500 ns x3	500 ns
12	p57	Cdk2	CyclinA	500 ns x3	500 ns
13	p57 Y2P91	Cdk2	CyclinA	500 ns x3	500 ns
14	p57 Y2P63	Cdk2	CyclinA	500 ns x3	500 ns
15	none	Cdk4	CyclinD	500 ns	500 ns
16	p27	Cdk4	CyclinD	500 ns	500 ns
17	p27 Y2P74	Cdk4	CyclinD	500 ns	500 ns
18	p21	Cdk4	CyclinD	500 ns	500 ns
19	p57	Cdk4	CyclinD	500 ns	500 ns
20	p57 Y2P63	Cdk4	CyclinD	500 ns	500 ns

Supplementary Table 2: CyclinA/Cdk2/p27 Intermolecular hydrogen bonds

Acceptor	Donor	Donor H	Fraction	Avg Dist	Avg Ang
Cdk2 GLU81 O	p27 TYR88 HH	p27 TYR88 OH	0.9277	2.7255	156.7414
Cdk2 LYS20 O	p27 GLN77 H	p27 GLN77 N	0.8497	2.8389	159.9591
p27 ARG30 O	CycA GLN254 HE21	CycA GLN254 NE2	0.7614	2.8550	163.6496
p27 GLN77 O	Cdk2 LYS20 H	Cdk2 LYS20 N	0.6941	2.8750	164.4870
Cdk2 VAL18 O	p27 VAL79 H	p27 VAL79 N	0.6316	2.8766	162.2807
CycA THR285 OG1	p27 ASN31 HD22	p27 ASN31 ND2	0.5990	2.8777	163.1120
p27 VAL79 O	Cdk2 VAL18 H	Cdk2 VAL18 N	0.5775	2.8694	160.0881
CycA GLU268 O	Cdk2 ARG150 HH12	Cdk2 ARG150 NH1	0.5554	2.8259	155.3670
CycA GLU269 O	Cdk2 ARG159 HH22	Cdk2 ARG150 NH2	0.5444	2.8241	158.6409
p27 ALA28 O	CycA TRP217 HE1	CycA TRP217 NE1	0.5438	2.8429	152.3201

Supplementary Table 3: CyclinA/Cdk2/p57 Intermolecular hydrogen bonds

Acceptor	Donor	Donor H	Fraction	Avg Dist	Avg Ang
Cdk2 GLU81 O	p57 TYR91 HH	p57 TYR91 OH	0.9252	2.7349	157.1118
Cdk2 LYS20 O	p57 THR80 H	p57 THR80 N	0.7832	2.8444	159.7401
p57 ARG31 O	CycA GLN254 HE21	CycA GLN254 NE2	0.7393	2.8585	163.2721
p57 VAL82 O	Cdk2 VAL18 H	Cdk2 VAL18 N	0.7028	2.8656	161.7094
p57 THR80 O	Cdk2 LYS20 H	Cdk2 LYS20 N	0.6906	2.8719	164.6074
Cdk2 VAL18 O	p57 VAL82 H	p57 VAL82 N	0.6027	2.8825	160.8764
CycA GLU268 OE2	Cdk2 ARG150 HH12	Cdk2 ARG150 NH1	0.5722	2.8099	157.1202
p57 ALA29 O	CycA TRP217 HE1	CycA TRP217 NE1	0.5302	2.8593	156.4832

Supplementary Table 4: CyclinA/Cdk2/p21 Intermolecular hydrogen bonds

Acceptor	Donor	Donor H	Fraction	Avg Dist	Avg Ang
Cdk2 GLU81 O	p21 TYR77 HH	p21 TYR77 OH	0.8877	2.7510	155.7238
Cdk2 LYS20 O	p21 GLU66 H	p21 GLU667 N	0.8584	2.8434	160.1049
p21 VAL68 O	Cdk2 VAL18 H	Cdk2 VAL18 N	0.7242	2.8612	161.6964
p21 ARG19 O	CycA GLN254 HE21	CycA GLN254 NE2	0.7212	2.8605	163.2194
Cdk2 VAL18 O	p21 VAL68 H	p21 VAL68 N	0.7042	2.8720	162.7763
p21 GLU66 O	Cdk2 LYS20 H	Cdk2 LYS20 N	0.6416	2.8808	165.1146
p21 ALA17 O	CycA TRP217 HE1	CycA TRP217 NE1	0.5339	2.8445	151.2931
p21 ASP62 O	Cdk2 ASN23 HD22	Cdk2 ASN23 ND2	0.5275	2.8400	153.8981

Supplementary Table 5: CyclinA/Cdk2 Intermolecular hydrogen bonds

Acceptor	Donor	Donor H	Fraction	Avg Dist	Avg Ang
CycA GLU269 O	Cdk2 ARG150 HH22	Cdk2 ARG150 NH2	0.6919	2.8348	159.3739

Supplementary Table 6: CyclinD/Cdk4/p27 Hbond details

Acceptor	Donor	Donor H	Fraction	Avg Dist	Avg Ang
p27 GLN77 O	Cdk4 LYS24 H	Cdk4 LYS24 N	0.8241	2.8484	162.4492
CycD ALA153 O	Cdk4 ARG61 HH11	Cdk4 ARG61 NH1	0.8058	2.8270	159.2312
p27 ARG30 O	CycD GLN100 HE21	CycD GLN100 NE2	0.7887	2.8483	163.3430
Cdk4 LYS24 O	p27 GLN77 H	p27 GLN77 N	0.7069	2.8672	162.2508
CycD LYS149 O	Cdk4 ARG60 HE	Cdk4 ARG60 NE	0.6619	2.8316	152.9677
Cdk4 ASP128 OD2	CycD ARG26 HE	CycD ARG26 NE	0.6271	2.8293	160.7100
Cdk4 ASP128 OD1	CycD ARG26 HH21	CycD ARG26 NH2	0.6240	2.8094	158.9971
CycD GLU141 OE2	Cdk4 LEU48 H	Cdk4 LEU48 N	0.5566	2.8669	162.4664

Supplementary Table 7: CyclinD/Cdk4/p57 Hbond details

Acceptor	Donor	Donor H	Fraction	Avg Dist	Avg Ang
p57 THR80 O	Cdk4 LYS24 H	Cdk4 LYS24 N	0.7715	2.8547	161.5388
CycD LYS149 O	Cdk4 ARG60 HH11	Cdk4 ARG60 NH1	0.7577	2.8222	158.0258
p57 ARG31 O	CycD GLN100 HE21	CycD GLN100 NE2	0.7568	2.8580	163.1346
Cdk4 ASP128 OD2	CycD ARG26 HH22	CycD ARG26 NH2	0.6672	2.7841	161.6650
Cdk4 ASP27 OD1	p57 ARG76 HH22	p57 ARG76 NH2	0.6490	2.7876	161.4593
Cdk4 ASP128 OD1	CycD ARG26 HH12	CycD ARG26 NH1	0.6158	2.8135	161.4324
Cdk4 LYS24 O	p57 THR80 H	p57 THR80 N	0.6010	2.8724	160.2666
Cdk4 ASP27 OD2	p57 ARG76 HH12	p57 ARG76 NH1	0.5057	2.8207	155.3517

Supplementary Table 8: CyclinD/Cdk4/p21 Hbond details

Acceptor	Donor	Donor H	Fraction	Avg Dist	Avg Ang
p21 GLU66 O	Cdk4 LYS24 H	Cdk4 LYS24 N	0.8738	2.8409	162.3939
p21 ARG19 O	CycD GLN100 HE21	CycD GLN100 NE2	0.8019	2.8466	163.5126
Cdk4 LYS24 O	p21 GLU66 H	p21 GLU66 N	0.6739	2.8733	161.0108
CycD LYS149 O	Cdk4 ARG60 HE	Cdk4 ARG60 NE	0.5691	2.8463	155.4499
Cdk4 ASP75 OD2	p21 TRP49 HE1	p21 TRP49 NE1	0.5166	2.8326	163.6698

Supplementary Table 9: CyclinD/Cdk4 Hbond details

Acceptor	Donor	Donor H	Fraction	Avg Dist	Avg Ang
Cdk4 ASP128 OD2	CycD ARG26 HH22	CycD ARG26 NH2	0.9339	2.7818	160.7384
Cdk4 ASP128 OD1	CycD ARG26 HH12	CycD ARG26 NH1	0.8858	2.8056	163.3988
CycD LYS149 O	Cdk4 ARG60 HE	Cdk4 ARG60 NE	0.6789	2.8408	156.3797
CycD GLU141 OE1	Cdk4 LEU48 H	Cdk4 LEU48 N	0.6267	2.8530	160.3516

Supplementary Movie 1: The molecular dynamics trajectories from CyclinA:CDK:p27 (PDB: 1JSU) and homology models of CyclinA:CDK in complex with p21 and p57 are shown in the top row, along with the conserved tyrosine phosphorylated inhibitor complexes (p27-Y88p/p21-Y77p/p57-Y91p) in the lower row. The intramolecular peptide backbone RMSD is shown highlighting the ejection of the 3_{10} helix in tyrosine phosphorylated p57 in cMD simulations. Also note the increased mobility of the C-lobe of CDK2 (at the top of the shown structures) in tyrosine-phosphorylated p27 and p57 complexes, but not in phosphorylated p21 trimers.

References

(1) Miao, Y.; Sinko, W.; Pierce, L.; Bucher, D.; Walker, R. C.; McCammon, J. A. Improved reweighting of accelerated molecular dynamics simulations for free energy calculation. *Journal of chemical theory and computation* **2014**, *10*, 2677–2689.

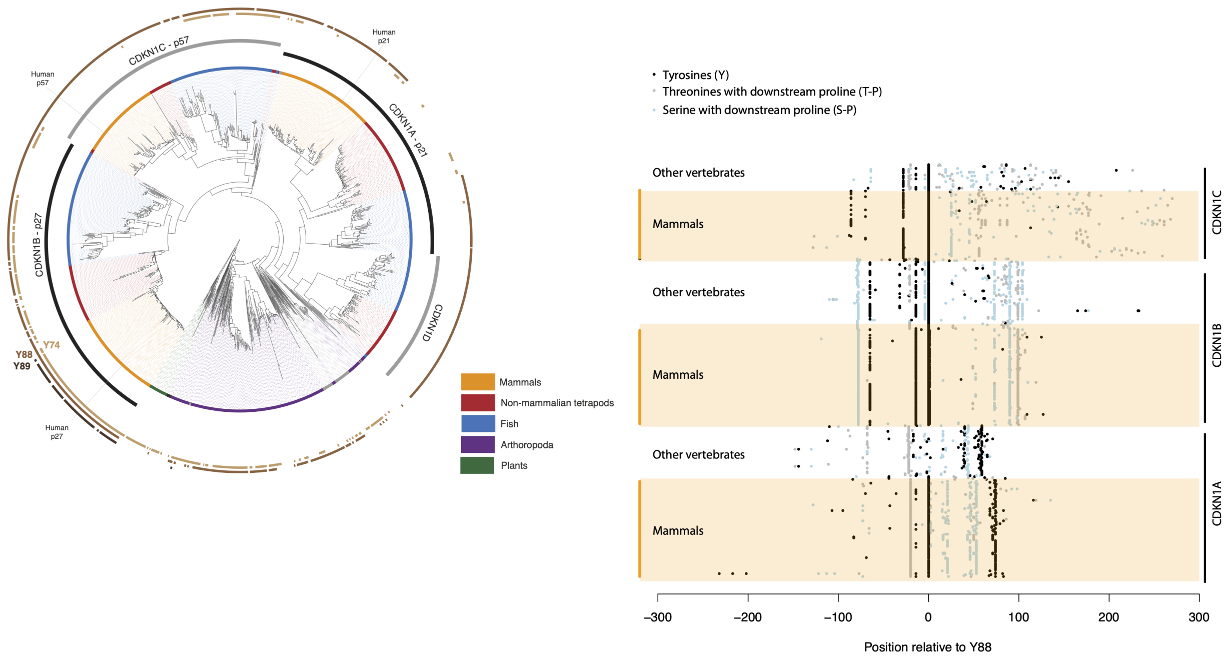

(a) (b)

Supplementary Figure 1: Phylogeny of the Cip/Kip family and conservation of residues subject to phosphorylation. (a) Protein-level maximum likelihood phylogeny of Cip/Kip family members across eukaryotes (see Methods for data provenance). Based both on prior annotation and the phylogenetic splits observed here, CdkN1A (p21), CdkN1B (p27), CdkN1C (p57), and CdkN1D (p20) sequences form coherent monophyletic clades and are labelled accordingly. The presence/absence of key tyrosine residues (Y74, Y88, Y89 in p27) across the tree is indicated. (b) Distribution of potential phosphorylation sites across members of the Cip/Kip family. Tyrosine = black; serine followed by a downstream proline = light blue; threonine followed by a downstream proline = grey. For simplicity, only orthologs previously explicitly annotated as CdkN1A, CdkN1B, or CdkN1C in mammals and non-mammalian tetrapods (birds, reptiles, amphibians) are considered here. Sequences were centred on orthologous position Y88 based on the protein alignment (see Methods). The distance to neighbouring phosphorylation sites was then computed for each sequence independently and therefore faithfully represents the distance along the protein primary sequence rather than along the (gapped) alignment.

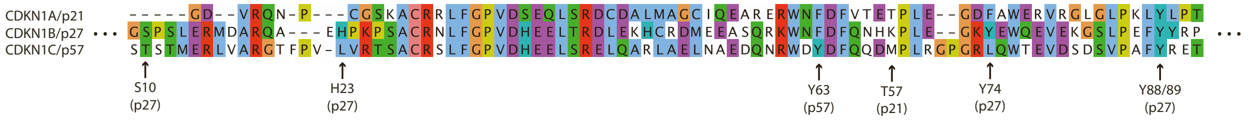

Supplementary Figure 2: Partial alignment of human p21, p27, and p57, extracted from the larger alignment of eukaryotic CIP/KIP orthologs (see main text Methods). Residues of particular interest are highlighted.

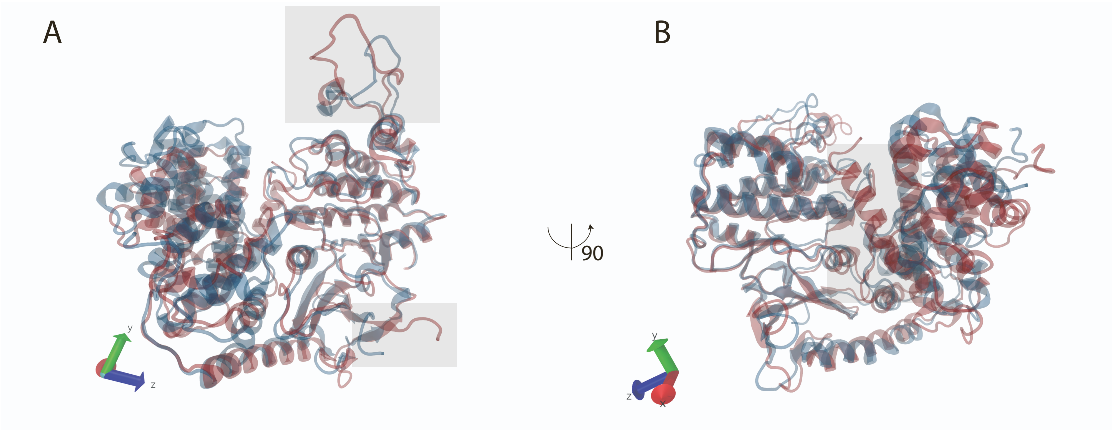

Supplementary Figure 3: CyclinA/Cdk2/p27 (blue) superimposed with CyclinA/Cdk2/p27^{Y2P74} (red) at 500 ns, and rotated by 90° in B. Change in RMSD can be attributed to the CyclinA alpha-helix position highlighted in subfigure B. Subfigure A highlights flexible C-terminal and loop regions of Cdk2.

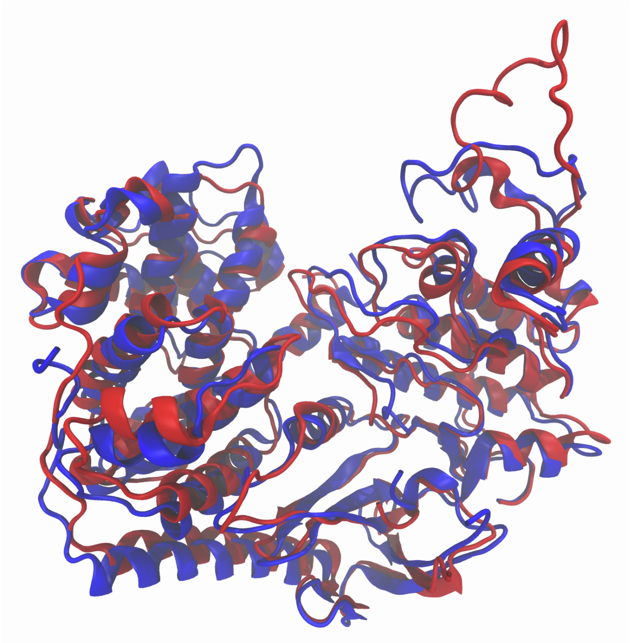

(a)

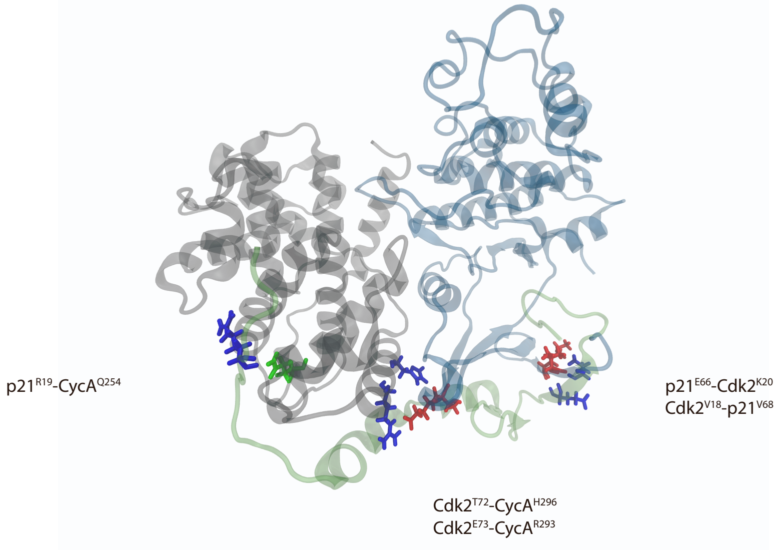

(b)

Supplementary Figure 4: (a) The conformation of CyclinA/Cdk2/p21 jumping from one state to another, happening at around 100 (blue) and 340 ns (red) and (b) CyclinA/Cdk2/p21 structure at 340 ns, where p21 has been phosphorylated at Y77. The structure at shows the addition of long-lasting intermolecular hydrogen bonding between all three molecules, not observed in the other complexes

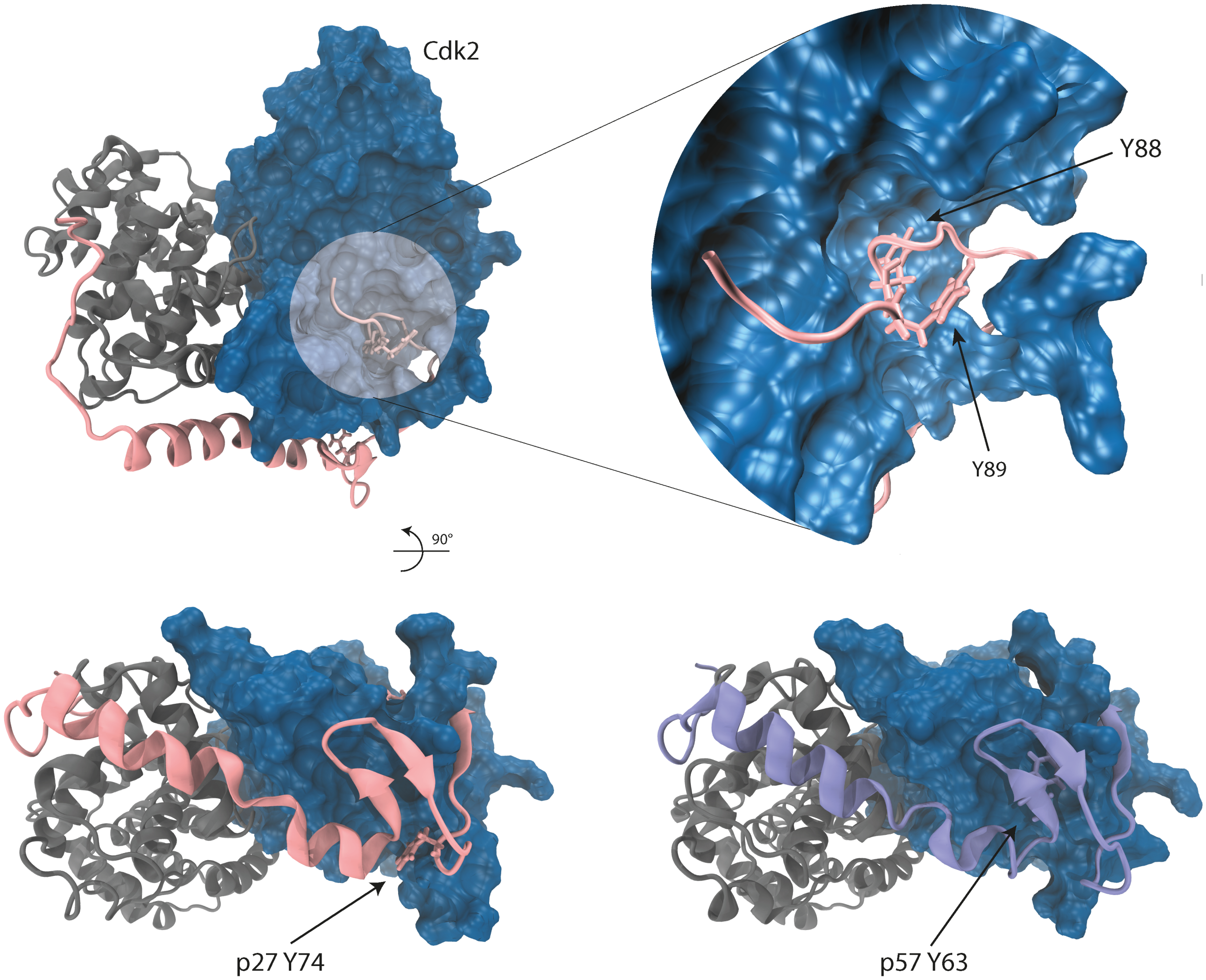

Supplementary Figure 5: Structure of CyclinA/Cdk2/p27 where Cdk2 is displayed as solvent accessible surface area. These structures highlight the inaccessible/buried nature of p27 Y88, hidden within the Cdk2 active site and obscured by the terminal end of Cdk2. The adjacent Y89 residue is more accessible and potentially more likely to be phosphorylated by a NRTK. The structures for p27 and p57 have been rotated in order to highlight the positions of p27 Y74 and p57 Y63.

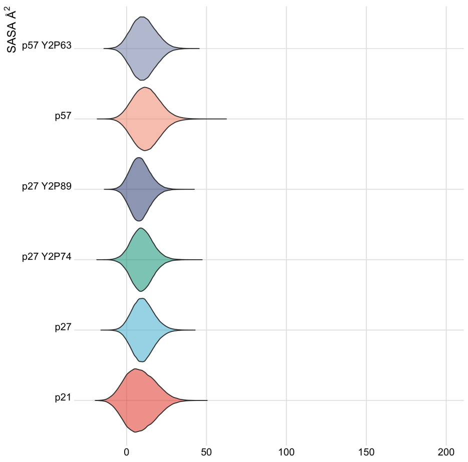

(a)

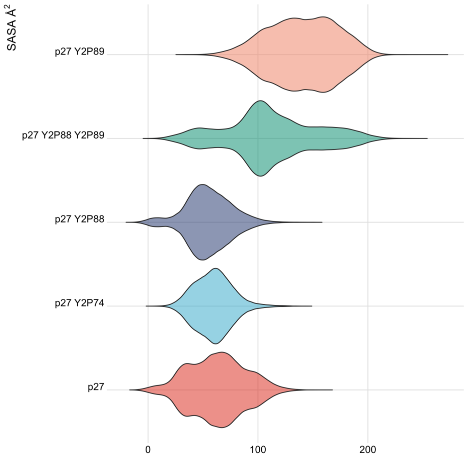

(b)

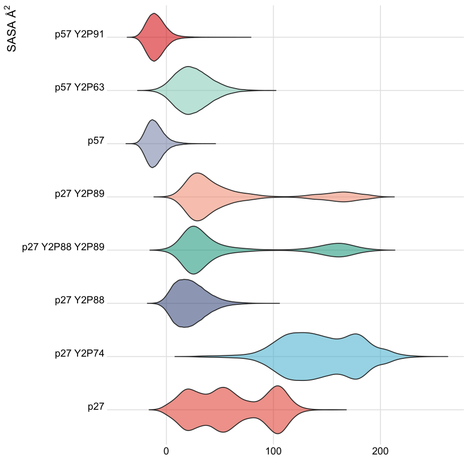

(c)

Supplementary Figure 6: Density of SASA of residue taken from three independent cMD simulations for each model.

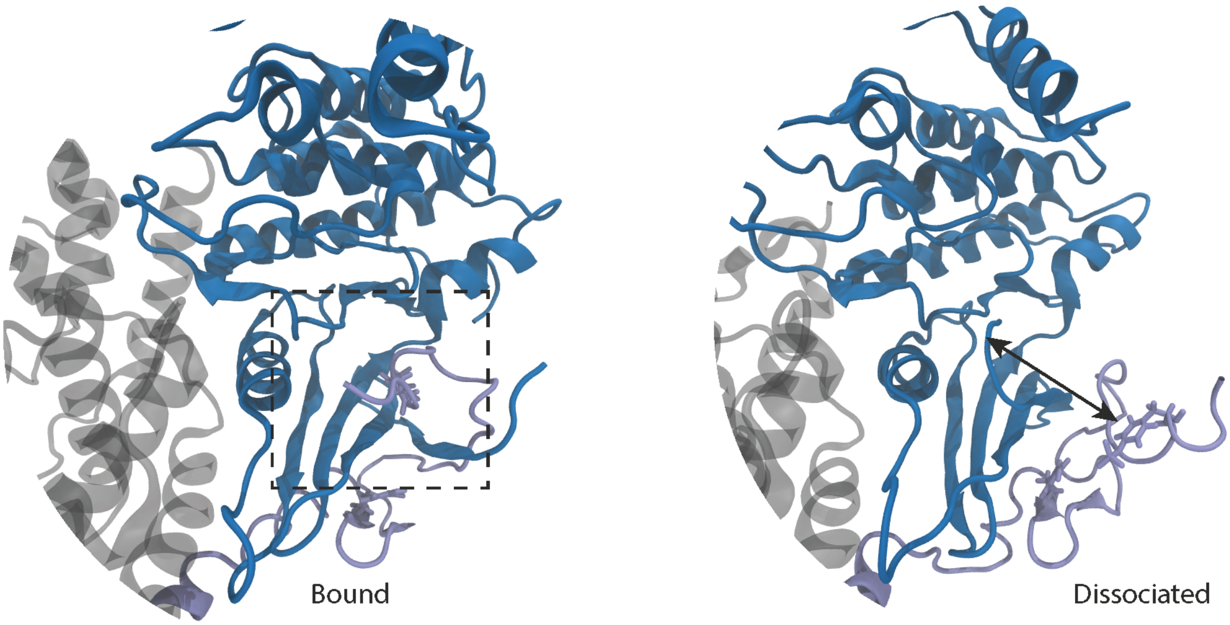

Supplementary Figure 7: The starting conformation of p57 bound to Cdk2/CyclinA, where we have highlighted the p57 Y91 residue tightly bound to the Cdk2 active site (left), and the final conformation from simulation highlighting the dissociation of p57 Y91 from Cdk2 and apparent reactivation of Cdk2.

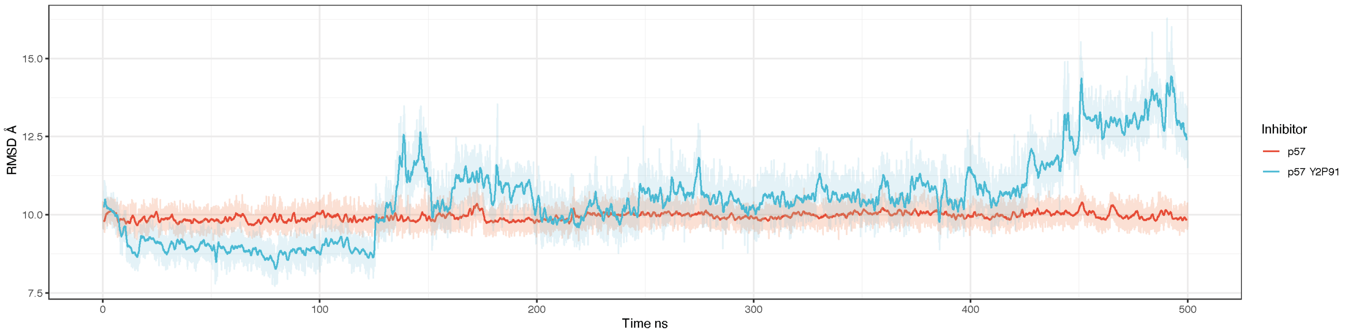

(a)

Supplementary Figure 8: Centre-of-mass distance between the 3_{10} α-helicies and the active site pocket for p57 from cMD. The only complex displaying dissociation of the helix from classical molecular dynamics.

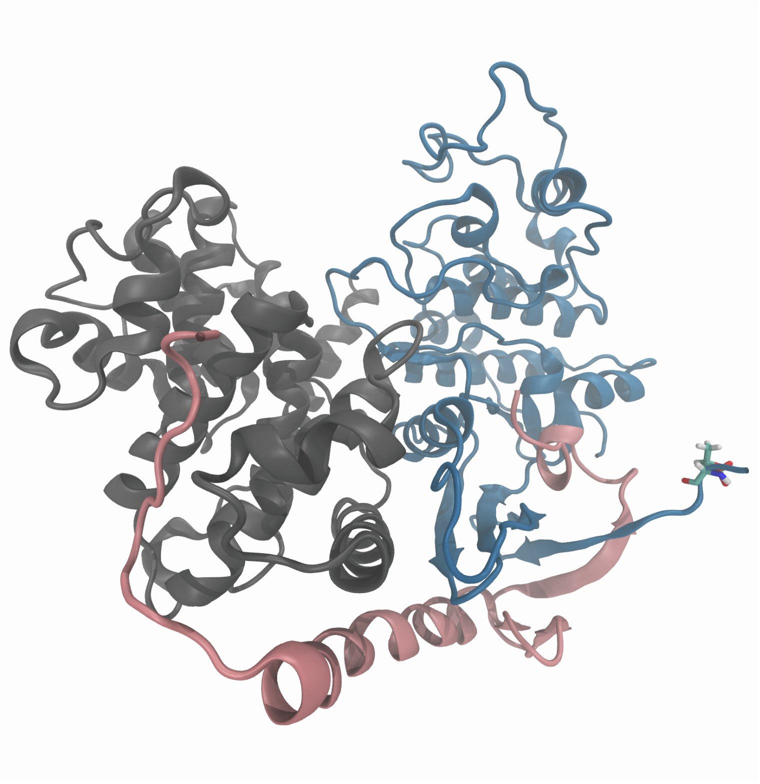

Supplementary Figure 9: Phosphorylation of T14 in Cdk2 makes the N-terminus of Cdk2 becomes more diffuse showing that it is no longer bound to p27

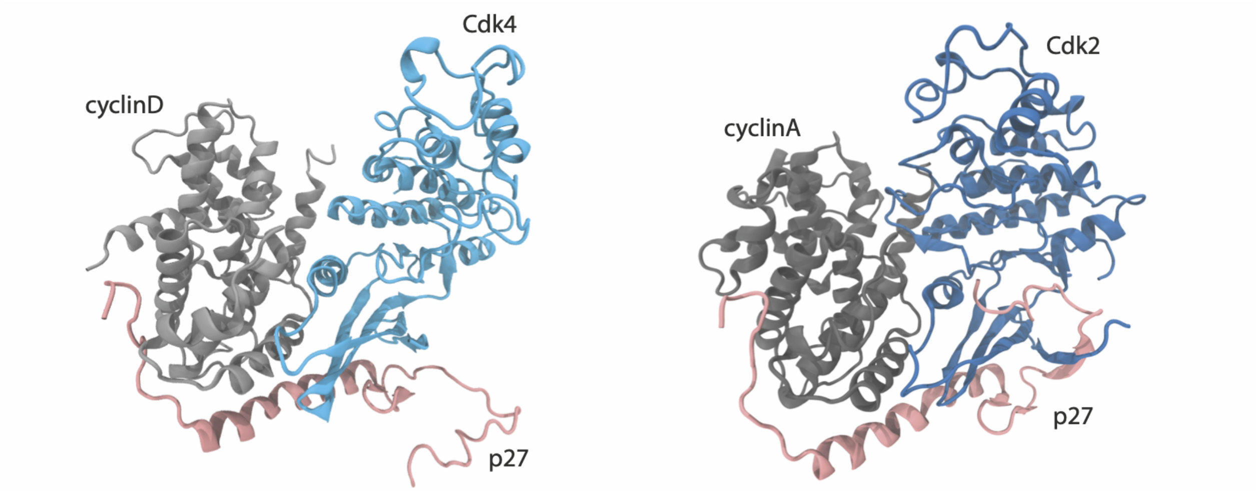

Supplementary Figure 10: A side-by-side comparison of the p27 bound Cdk2/CyclinA and Cdk4/CyclinD complexes.

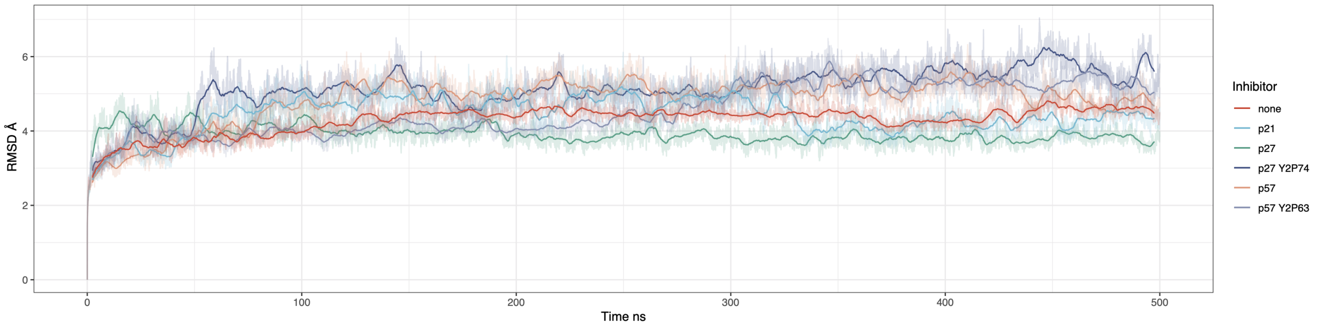

(a) Whole system

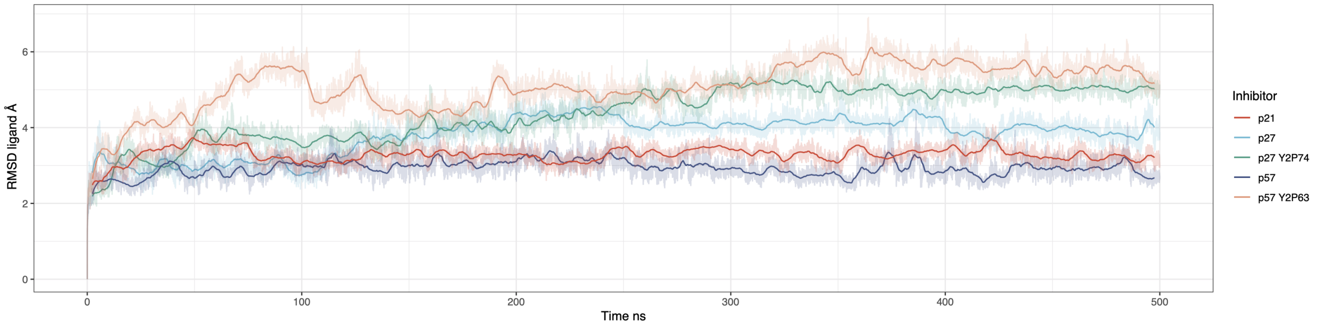

(b) Inhibitor only

Supplementary Figure 11: Root mean squared deviation from the crystal structure after minimisation and heat-up for each model of (a) the full complex and (b) the inhibitor. The p27 Y2P74 model displays greater deviation from it's starting structures than other models.

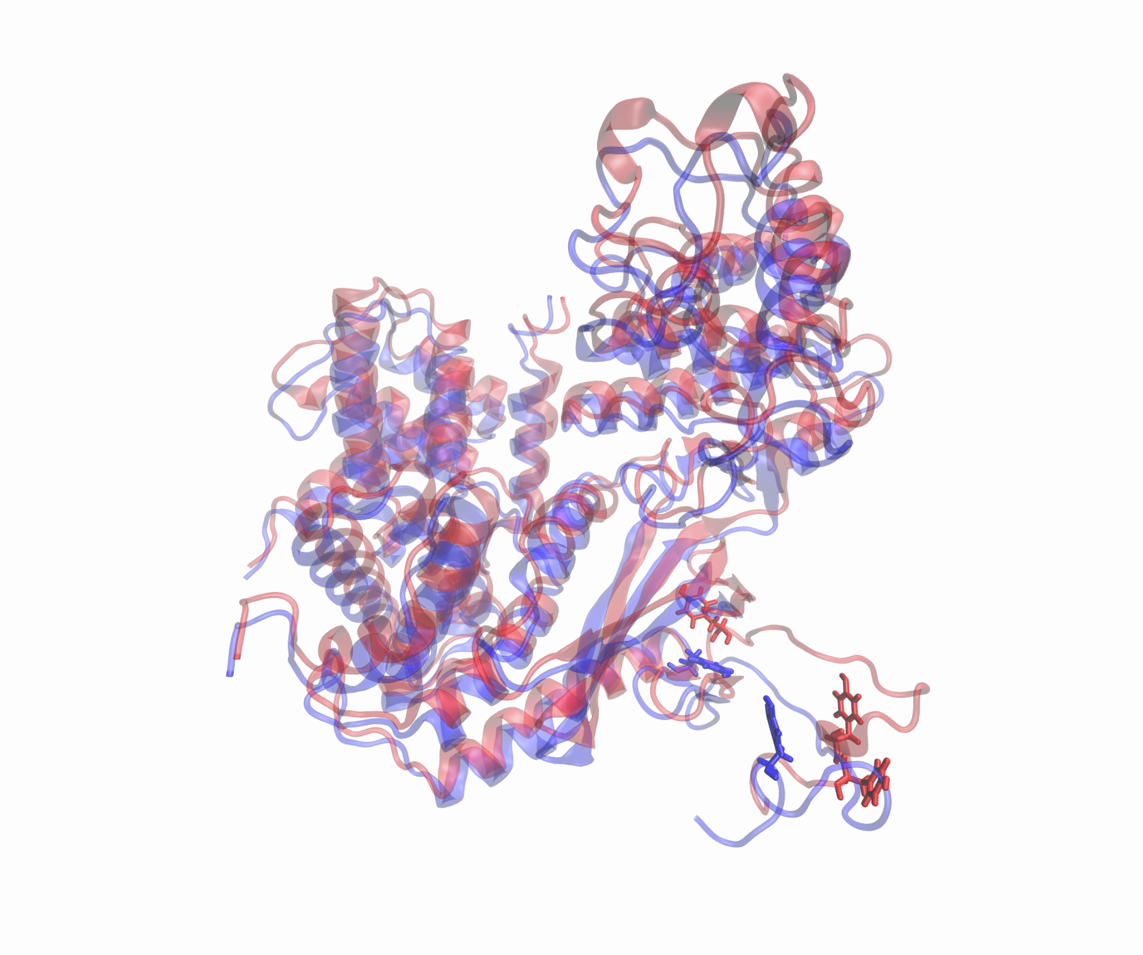

Supplementary Figure 12: p27 (red) and p57 (blue) bound Cdk4/CyclinD, with CKI tyrosine positions shown.
